# GSK3β palmitoylation mediated by ZDHHC4 promotes tumorigenicity of glioblastoma stem cells in temozolomide-resistant glioblastoma through the EZH2–STAT3 axis

**DOI:** 10.1038/s41389-022-00402-w

**Published:** 2022-05-23

**Authors:** Chenggang Zhao, Huihan Yu, Xiaoqing Fan, Wanxiang Niu, Junqi Fan, Suling Sun, Meiting Gong, Bing Zhao, Zhiyou Fang, Xueran Chen

**Affiliations:** 1 Anhui Province Key Laboratory of Medical Physics and Technology; Institute of Health and Medical Technology, Hefei Institutes of Physical Science, Chinese Academy of Sciences, No. 350, Shushan Hu Road, 230031 Hefei, Anhui China; 2 Science Island Branch, Graduate School of University of Science and Technology of China, No. 96, Jin Zhai Road, 230026 Hefei, Anhui China; 3 Department of Laboratory Medicine, Hefei Cancer Hospital, Chinese Academy of Sciences, No. 350, Shushan Hu Road, 230031 Hefei, Anhui China; 4 MOE Key Laboratory for Membraneless Organelles and Cellular Dynamics, University of Science & Technology of China, No. 96, Jin Zhai Road, 230027 Hefei, Anhui China; 5 Department of Anesthesiology, The First Affiliated Hospital of USTC, Division of Life Sciences and Medicine, University of Science and Technology of China (USTC), No. 17, Lu Jiang Road, 230001 Hefei, Anhui China; 6 Department of Neurosurgery, The Second Affiliated Hospital of Anhui Medical University, No. 678, Fu Rong Road, 230601 Hefei, Anhui China

**Keywords:** CNS cancer, Post-translational modifications, Tumour biomarkers

## Abstract

Glioblastoma stem cells (GSCs) are a highly tumorigenic cell subgroup of glioblastoma (GBM). Glycogen synthase kinase 3β (GSK3β) is considered a key hub for promoting malignant phenotypes in GBM. However, the functional relationships between GSK3β and GSCs in GBM are unclear. Here, we found that GSK3β was noted as a substrate for ZDHHC4-mediated palmitoylation at the Cys14 residue, which enhanced GBM temozolomide (TMZ) resistance and GSC self-renewal. Clinically, the expression level of ZDHHC4 was upregulated in GBM, which significantly correlated with tumor grade and poor prognosis. The above phenotypes were based on decreasing *p-*Ser9 and increasing *p-*Tyr216 by GSK3β palmitoylation, which further activated the enhancer of the zeste homolog 2 (EZH2)–STAT3 pathway. Notably, STAT3 silencing also inhibited ZDHHC4 expression. This study revealed that GSK3β palmitoylation mediated by ZDHHC4 improved the stemness of TMZ-resistant GBM by activating the EZH2–STAT3 signaling axis, providing a new theoretical basis for further understanding the mechanism of TMZ resistance and recurrence after treatment.

## Introduction

A glioblastoma (GBM) is one of the most common primary tumors in the adult central nervous system [1]. The standard therapy for GBM is surgery, radiotherapy, and chemotherapy, and has only limited effectiveness. Due to the blood-brain barrier, temozolomide (TMZ) has become the most commonly used chemotherapy drug in clinics [2]. Glioblastoma stem cells (GSCs), conceptually defined as a population of tumor cells capable of self-renewal and multipotency, are important players that contribute to GBM chemotherapy resistance [3]. This results in a significant impact on tumor cell plasticity, intratumor heterogeneity, invasion and metastasis, therapeutic resistance, and tumor recurrence after surgery and adjuvant therapy [3–5]. Therefore, the eradication of GSCs is of great clinical significance in the fight against refractory cancer.

To date, various molecular mechanisms have been implicated in maintaining CSC self-renewal [5]. Numerous studies have identified GSK3β, a serine/threonine protein kinase that contributes to the maintenance of the GSC phenotype. The earliest proven function of GSK3 is glycogen synthase inactivation [6]. GSK3 has two subtypes, α and β, in mammals. The proteins GSK3α and GSK3β are encoded by different genes. The kinase catalytic regions of the two subtypes are 98% structurally similar, and they are mainly distinguished from the N-terminal domain [7, 8]. GSK3β is involved in tumor formation and progression and has been suggested as a tumor suppressor because its activation accelerates the degradation of oncogenes such as β-catenin, cyclin D1, and c-Myc [9]. However, it also promotes cell proliferation and survival in patients with advanced tumors [10]. Phosphorylation at tyrosine 216 of the GSK3β protein is a key indicator of its activity. In contrast, phosphorylation of serine 9 inhibits its kinase activity [8, 11]. The upstream signals that negatively regulate GSK3β activity include p70S6K, ERK, AKT, PKC, PKA, and p90RSK, which phosphorylate ser9 and cause GSK3β inactivation [10, 11]. Many studies have provided a clear direction for the function of GSK3β in GSCs [12–14]. In addition, as an important transcription factor, STAT3 plays an important role in maintaining GSC self-renewal [15]. GSK3β and STAT3 are two molecular markers that have been identified to regulate CSC activity, but their interactions are not clear.

Palmitoyl modification mediated by the ZDHHC family has been found to play an important role in the development of cancer. Palmitoylation is one of the protein post-translational modifications in which a 16-carbon fatty acid palmitate is covalently bound to a protein-specific cysteine (Cys) residue via a thioester bond. Palmitoylation can regulate protein stability, subcellular localization, protein vesicular transport, and other intracellular life processes. Currently, the functions of many cancer-related proteins (RAS, EGFR, STAT3, etc.) have been found to be regulated by palmitoylation modification [16–18]. ZDHHC4, a member of the ZDHHC family, has recently been shown to regulate the palmitoylation of KAI1 to localize it to the cell membrane, thereby inhibiting angiogenesis [19]. However, the role and mechanism of ZDHHC4 in the development of tumors, especially GBM, have not yet been elucidated. Here, we found that GSK3β can be palmitoylated by ZDHHC4 to change the phosphorylation level, thus activating the EZH2/STAT3 axis to promote GSC tumorigenicity and ultimately enhance GBM TMZ-resistance.

## Materials and methods

### Antibodies and reagents

Antibodies against GSK3β (12456), *p-*GSK3β (S9) (8566), GSK3α (4337), *p-*GSK3α (S21) (9316), EZH2 (5246), STAT3 (9139), *p-*STAT3 (Y705) (9145), β-catenin (8480), AKT1 (4691), and HA (3724) were purchased from Cell Signaling Technology. Antibodies against GFP (50430), β-tubulin (66031), PKA (55382), p70S6K (14485), p90RSK (16463), and MGMT (17195) were purchased from Proteintech (Wuhan, China). ZDHHC4 (ab235369), *p-*GSK3α + β (Y216 + Y279) (ab68476), and *p-*EZH2 (S21) (ab84989) were obtained from Abcam. Antibodies against FLAG (F1804) were purchased from Sigma–Aldrich. Methyl K. AlexaFluor488 goat anti-rabbit IgG (A11008) and AlexaFluor568 goat anti-rabbit IgG (A11011) were purchased from Invitrogen (Carlsbad, CA, USA). Rhodamine phalloidin (#PHDR1) was purchased from Cytoskeleton (USA). Protein chips were obtained from R&D Systems (USA).

TMZ (HY-17364) and Niclosamide (HY-B0497) were purchased from MedChemExpress (Shanghai, China). N-ethylmaleimide (NEM) (23030), hydroxylamine HCl (HAM) (26103), and EZ-Link™ HPDP-Biotin (A35390) were obtained from Thermo Fisher. DMSO (D2650) was purchased from Sigma–Aldrich.

### Plasmid constructs, siRNA, and stable cell line generation

HA-GST and 23 HA-ZDHHC plasmids were gifts from M. Fukata (National Institute for Physiological Sciences). pcDNA3.1-Flag-GSK3β, pcDNA3.1-Flag-ZDHHC4, pcDNA3.1-Flag-STAT3, and lentiviral shRNA plasmids were purchased from Public Protein/Plasmid Library (Nanjing, China). GSK3β cDNA and ZDHHC4 cDNA were cloned into the pEGFP vector to construct the pEGFP-GSK3β and pEGFP-ZDHHC4 plasmids.

All point mutation plasmids GSK3β (C14A), GSK3β (S9A), GSK3β (Y216A), EZH2 (S21A), and ZDHHC4 (C179S) were obtained by designing primers according to the reverse overlap method. Using the wild-type plasmid as a template, the mutant was amplified using a FastPfu Fly DNA Polymerase kit (AS231-01, TransGen Biotech, Beijing, China). The primer sequences are listed in Supplementary Table S1.

pLKO.1-ZDHHC4 and pLVX-ZDHHC4 plasmids were obtained from Origene (Wuxi, China). Flag-labeled GSK3β (WT) cDNA and GSK3β (C14A) cDNA were cloned into lentiviral vectors (pCDH-EF1-FHC-puro, FENGHUISHENGWU, Wuhan, China) for the expression of constituent genes. To establish cell lines stably overexpressing GSK3β (WT) and GSK3β (C14A), the pCDH-EF1-FHC-GSK3β (WT) and pCDH-EF1-FHC-GSK3β (C14A) plasmids were co-transfected with psPAX2 + pMD2.G plasmids into 293 T cells for 48 h to obtain a virus superfluid. SF126R and U118R (R indicates TMZ resistance) cells were infected with the virus particles for 48 h. Stably transduced cells were screened by adding puromycin (500 ng/ml).

All siRNAs were synthesized by GenePharma (Shanghai, China). The shRNA and siRNA sequences are listed in Supplementary Tables S2 and S3.

For all transient transfections, plasmids or siRNAs were transfected into cells for 24/48 h using the Lipofectamine 2000 (11668019, Invitrogen) according to the manufacturer’s instructions.

### Cell culture

All glioma cells (SF126, U118MG, SW1088, SW1783, A172, LN18, T98G, and H4) used in this study were obtained from Cellcook (Guangzhou, China), and STR certificates for each cell line were provided. All cell lines were cultured with DMEM (Gibco, USA) containing 10% fetal bovine serum (HyClone, USA).

### Immunoblotting and immunoprecipitation

Cells were lysed using RIPA buffer (50 mM Tris [pH 7.4], 150 mM NaCl, 1% Triton X-100, 1% sodium deoxycholate, 0.1% SDS, sodium orthovanadate, sodium fluoride, EDTA, and leupeptin) (Beyotime, Shanghai, China) with a cocktail (Roche). Lysates were boiled at 100 °C for 10 min and then subjected to SDS-PAGE. The separated samples were transferred onto a nitrocellulose (NC) membrane. The membrane was blocked with 5% milk in PBST at room temperature for 1 h and then incubated with the corresponding primary antibody at 4 °C overnight. The membrane was incubated with horseradish peroxidase-conjugated secondary antibodies at room temperature for 1 h. Chemiluminescence substrates (NCM Biotech, Suzhou, China) were used to visualize the western blots.

For immunoprecipitation, western blot and IP lysates (20 mM Tris [pH 7.5], 150 mM NaCl, 1% Triton X-100, sodium pyrophosphate, β-glycerophosphate, EDTA, Na3VO4, and leupeptin) (Beyotime, Shanghai, China) with a cocktail (Roche) were used to prepare protein extracts. One microgram of the corresponding antibody was added to every 500 µg lysate and combined overnight at 4 °C. The mixture was incubated with 30 µL of protein A/G beads at room temperature for 2 h. After the bonded beads were washed with PBST five times, 30 µL SDS sample buffer was added for western blotting analysis.

### RNA extraction and quantitative real-time PCR

Total RNA was extracted using TRIZOL (Invitrogen). First-strand cDNA Synthesis SuperMix (TransGen Biotech, Beijing, China) was used to synthesize cDNA. RT-PCR was performed using the QuantiNova SYBR Green PCR Kit (Qiagen, Germany) according to the manufacturer’s instructions. RT-PCR analysis was performed using a LightCyker480 PCR machine (Roche) in triplicate. mRNA expression was calculated using the 2^−∆∆CT^. The primer sequences used for PCR are listed in Supplementary Table S4.

### Tumor sphere formation and extreme limiting dilution assay (ELDA)

Neurosphere formation experiments were performed as previously described [20]. Briefly, the cells were separated into individuals and inoculated with a total of 5000 cells. The 50 µm cell cluster was regarded as a neurosphere, photographed, and trypsinized to cultivate the second generation.

For the ELDA experiment, cells were seeded at 1, 5, 10, 20, and 25 cells/well, with 15 replicates per group. When a 50 µM cloning sphere was formed, the experiment was terminated and counted under a microscope. The sphere formation rate and significance were analyzed using ELDA software (https://bioinf.wehi.edu.au/software/ELDA/).

### Cell proliferation assay

CCK-8 assay (Cell Counting Kit-8, Beyotime, Shanghai, China) was used to detect cell viability. In a 96-well plate, 3000 cells were inoculated into each well and repeated for five wells per group.

Cell counting was performed to assess cell proliferation. A total of 2 × 10^5^ cells were inoculated into each culture. After treatment with different concentrations of TMZ, the cells were digested and resuspended for counting.

For the colony formation assay, 500 cells/well were seeded in a six-well plate. The medium mixed with the drugs was replaced every 2 days. After 2 weeks, the cells were fixed with 4% paraformaldehyde and stained with 0.1% crystal violet.

### Acyl-biotin exchange (ABE)

Cells were lysed using RIPA buffer. The obtained protein was incubated with 1 µg of antibody and NEM (alkylated free cysteine residues) at a final concentration of 50 mM at 4 °C overnight. On the second day, the complex was combined with protein A/G beads at room temperature for 1 h. Thereafter, 500 µL of 1 M HAM (pH 7.4) was added to the beads for 1 h at room temperature to cut the thioester bond. Five hundred microliters of 50 µM HPDP-Biotin was used to label the exposed sulfhydryl groups at 4 °C for 3 h. The beads were resuspended in 30 µL SDS sample buffer without reducing agents such as β-mercaptoethanol, boiled at 100 °C for 10 min, and subjected to western blot analysis.

### Isolation and mass spectrometry of biotinylated proteins

GBM tissue was lysed, and divided into two fractions. In the HAM + sample, the palmitate residue was cleaved and exchanged with biotin. The HAM− condition served as a negative control. After the ABE reaction was completed, streptavidin beads were used to enrich the biotinylated proteins. Proteins enriched under HAM+ and HAM− conditions were identified using mass spectrometry. Proteins with at least two-fold greater abundance in the HAM+ sample were considered to be candidate proteins. Using this approach, we identified 32 palmitoylated proteins, and these putative palmitoylation proteins were validated using the CSS-Palm version 4.0 software.

### Antibody microarray analysis

Human Phospho-Kinase Array Kit (ARY003B) was purchased from R&D Systems, and antibody microarray analysis according to the manufacturer’s instructions. Add 200–600 µg of cell lysate to each Array unit. Block the protein chip membrane with 1 mL Array Buffer and incubate for 1 h. Add 1 mL of samples diluted with Array Buffer to each membrane and incubate at 4 °C overnight. Incubate the membrane with the detection antibody for 2 h at room temperature. Membranes were incubated with streptavidin-HRP for 30 min. Add 1 mL of chemical color reagent to each well, and use a chemiluminescence instrument to develop color.

### Immunofluorescence and immunohistochemistry

Cells were inoculated in a 12-well plate with the cell livers laid. Immunofluorescence detection was performed when the cells were 50% confluent. The cells were fixed with 4% paraformaldehyde and incubated with antibodies shown in the figure. Cells were then imaged using an inverted microscope (Leica Inverted Microscope).

For immunohistochemical staining of the glioma tissue chip (OUTDO BIOTECH, Shanghai, China), a series of xylene deparaffinization and alcohol rehydration followed by antigen retrieval (10 mM sodium citrate) were used. The antibodies used for staining were the same as those used for western blotting. To quantify the expression of ZDHHC4, *p-*GSK3β (Y216), and *p-*STAT3 (Y705), IHC staining was quantified using scores of 0 (0%), 1 (0–10%), 2 (10–20%), 3 (20–30%), 4 (30–40%), 5 (40–50%), 6 (50–60%), 7 (60–70%), 8 (70–80%), 9 (80–90%), and 10 (90–100%).

### Xenograft studies

Female BALB/c-A nude mice at 4–5 weeks of age were purchased from Charles River (Beijing, China). For intracranial xenograft tumors, 5 × 10^5^ U118R cells (shNC or shZDHHC4) in 5 µL PBS were injected into the right frontal lobe of nude mice. In the animal experiment of TMZ treatment, 3 days after tumor cell transplantation, intraperitoneal injection of DMSO or TMZ was performed. TMZ (25 mg kg^−1^ d^−1^) or DMSO was injected daily for 30 days. After 45 days, the entire brain tissue was collected, perfused with 4% formalin, and fixed overnight. Paraffin-embedded sections were stained with hematoxylin and eosin. To calculate the tumor volume, we measured the maximum area of each tumor and calculated the tumor volume as length × width^2^ × 0.52. The time of natural death of mice was recorded, and survival curves were plotted according to the Kaplan–Meier method.

### Bioinformatics and statistical analyses

Gene expression data from The Cancer Genome Atlas (TCGA) were obtained and analyzed using Gliovis (http://gliovis.bioinfo.cnio.es/). Glioma patient survival data from REMBRANDT, The Chinese Glioma Genome Atlas (CGGA), and The Cancer Genome Atlas (TCGA) were obtained and analyzed using Gene Expression Profiling Interactive Analysis (GEPIA2). Kaplan–Meier survival curves of GBM patients with low or high ZDHHC4 mRNA expression were used to evaluate patient prognosis.

The two-tailed *t*-test was used to assess the statistical differences between the two groups. Multiple group comparisons were performed using one-way analysis of variance. Kaplan–Meier survival curves were generated using GraphPad Prism 8 software, and significance tests were performed using the logarithmic rank-sum test. For ELDA, the significance of the difference between the indicated groups was determined using the χ^2^ test. The Pearson correlation test was used to analyze the correlation between ZDHHC4 expression and other proteins in the tissues. All statistical analyses are shown as mean ± SD. The number of repetitions for each experiment is shown in the legend. Statistical significance was set at *p* < 0.05.

## Results

### GSK3β palmitoylation mediated by ZDHHC4 inhibits its Ser9 phosphorylation activity

**Fig. 1:**
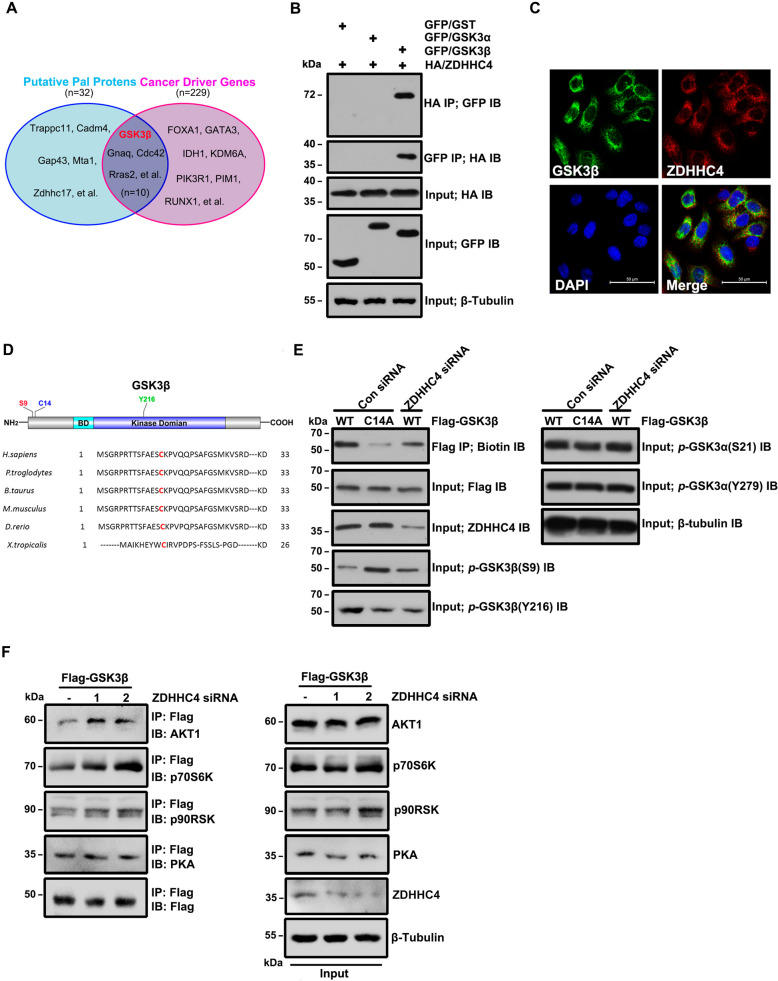
ZDHHC4-mediated palmitoylation alters GSK3β phosphorylation. **A** The collection of palmitoylated proteins and cancer driver genes in GBM. **B** The interaction of HA-ZDHHC4 with GFP-GSK3α and GFP-GSK3β was verified by immunoprecipitation in 293 T cells. **C** The localization of ZDHHC4 and GSK3β in SF126 cells was detected by immunofluorescence staining. **D** Protein map showing the three post-translationally modified amino acid residues in GSK3β. **E** SF126 cells were transfected, and the experiment was divided into three groups: wild-type Flag-GSK3β, C14A mutant GSK3β, and wild-type Flag-GSK3β were simultaneously knocked down ZDHHC4 by siRNA. ABE analysis and phosphorylation of GSK3α and GSK3β were performed in the three groups. The experiment was repeated twice. **F** ZDHHC4 was knocked down by siRNA in SF126 cells with stable expression of Flag-GSK3β. Immunoprecipitation assays showed the interaction of Flag-GSK3β with AKT, p70S6K, PKA, and p90RSK.

We isolated palmitoylated proteins from GBM cells with ABE and identified many cancer-driving genes [21] using mass spectrometry (Supplementary Table S5), among which GSK3β has not been reported (Fig. 1A). To determine which ZDHHC family members are responsible for the palmitoylation of GSK3β, we co-transfected 23 ZDHHC palmitoyl transferases with hemagglutinin (HA)-tagged 293 T cells together with GFP-GSK3β. ZDHHC4 was found to be the only enzyme that modified GSK3β (Supplementary Fig. 1A). Further analysis showed that GSK3β (but not GSK3α) interacted (Fig. 1B) and colocalized (Fig. 1C) with ZDHHC4. Cys14 was predicted to be a potential palmitoylation site of GSK3β using CSS-Palm 4.0 software, and Cys14 was evolutionarily conserved (Fig. 1D). We identified palmitoylation of GSK3β using ABE and found that both the GSK3β mutant (cysteine 14 mutated to alanine) and the siRNA silencing of ZDHHC4 made GSK3β palmitoylation significantly reduced in SF126 GBM cells, similar to the U118MG cell line (Fig. 1E; Supplementary Fig. 1B, C). GSK3β palmitoylation could be increased or decreased by transfection of wild-type GFP-ZDHHC4 (WT) or enzyme active site mutant GFP-ZDHHC4 (C179S) alone in SF126 and U118MG cells (Supplementary Fig. 1D, E).

Mutated GSK3β or ZDHHC4 silencing both enhanced *p-*GSK3β (S9) and inhibited *p-*GSK3β (Y216) but did not affect GSK3α phosphorylation (Fig. 1E; Supplementary Fig. 1C). GSK3β activity is negatively regulated by several upstream kinases. Based on this fact, it is reasonable to speculate that ZDHHC4 may competitively bind GSK3β with these kinases, leading to the continuous activation of GSK3β. A proximity ligation assay showed that ZDHHC4 was bound to p70S6K, AKT1, PKA, and p90RSK (Supplementary Fig. 2A). Further immunoprecipitation experiments showed that when ZDHHC4 was silenced, the binding ability of Flag-GSK3β with AKT1 and p70S6K (but not PKA and p90RSK) was improved. In contrast, compared with the control group, the binding ability of GFP-ZDHHC4 with AKT1 and p70S6K (but not PKA and p90RSK) of SF126 cells stably expressing Flag-GSK3β decreased (Fig. 1F; Supplementary Fig. 2B). Furthermore, we used the molecular simulation software Schrodinger Suites 2020.3 to predict the docking situation of GSK3β and the kinases. The results show that ZDHHC4, AKT1, and p70S6K (but not p90RSK and PKA) dock to the N-terminus of GSK3β protein (containing Ser9 and Cys14 residues). More detailed analysis showed that ZDHHC4 may bind to Phe10 and Lys15 of GSK3β through hydrogen bonds, to Met1, Arg4, Ser9, Cys14, and Lys15 of GSK3β through salt bridges, to Gly3 through disulfide bonds, and to Ser9, Ser13, and Lys15 through van der Waals forces (Supplementary Fig. 2C, Supplementary Table S6). RNAi-induced silencing of AKT and p70S6K both reduced p-GSK3β (S9), suggesting that AKT and p70S6K regulate p-GSK3β (S9) (Supplementary Fig. 2D). Taken together, these data suggest that ZDHHC4 may affect the binding of GSK3β to AKT1 and p70S6K, thereby inhibiting GSK3β Ser9 phosphorylation.

### GSK3β palmitoylation activates STAT3 by interacting with EZH2

**Fig. 2:**
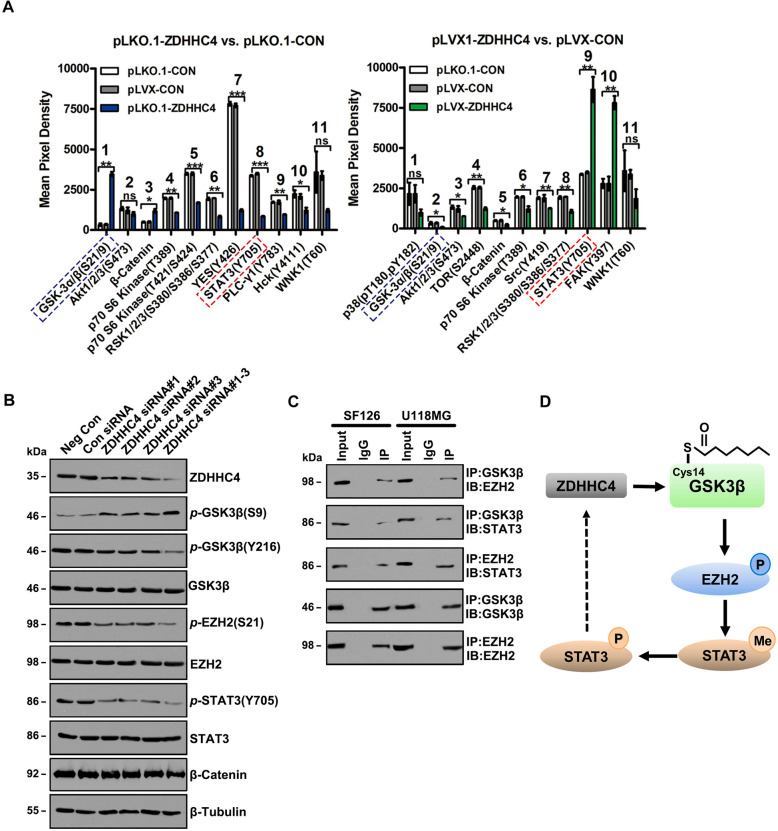
GSK3β palmitoylation regulates the EZH2–STAT3 axis. **A** Statistical analysis of Figure A showed a positive correlation between *p-*STAT3 (Y705) level and ZDHHC4 expression. Data are shown as means ± SD (*n* = 3). *P*-values were determined by two-tailed Student’s *t*-test. *P < 0.05; **P < 0.01; ***P < 0.001. **B** Western blot analysis of the phosphorylation activity of GSK3β, EZH2, and STAT3 after treatment with ZDHHC4 siRNA. **C** Immunoprecipitation was used to detect the interaction between GSK3β, EZH2, and STAT3 in SF126 and U118 cells. **D** Schematic diagram of GSK3β regulating the EZH2–STAT3 axis.

To further study the regulatory mechanism of GSK3β palmitoylation on downstream signals, protein microarray analysis showed that *p-*GSK3α/β (S21/S9) was negatively correlated with ZDHHC4 expression, whereas *p-*STAT3 (Y705) was positively correlated with ZDHHC4 expression in SF126 cells (Fig. 2A; Supplementary Fig. 3A-B). Silencing of ZDHHC4 in SF126 cells enhanced *p-*GSK3β (S9) and decreased *p-*GSK3β (Y216), *p-*EZH2 (S21) (EZH2 methylates and activates STAT3), and *p-*STAT3 (Y705) (Fig. 2B; Supplementary Fig. 3C). Although protein microarray screening revealed an inverse correlation between β-catenin and ZDHHC4 expression (Fig. 2A; Supplementary Fig. 3A, B), cytological experimental validation showed no change in β-catenin levels (Fig. 2B), indicating that GSK3β palmitoylation regulates signal transduction independent of β-catenin.

Next, we focused on detecting the regulation of STAT3 signaling by ZDHHC4. GSK3β, EZH2, and STAT3 interactions were found in both GBM cell lines (Fig. 2C) and were attenuated by GSK3β (C14 A) (Supplementary Fig. 4A). Overexpression of the activating mutant plasmid GSK3β (S9A) increased the phosphorylation of GSK3β (Y216), EZH2 (S21), and STAT3 (Y705) and the methylation of STAT3, while GSK3β (C14A), GSK3β (Y216A), and EZH2 (S21A) had the opposite effect (Supplementary Fig. 4A-D). Interestingly, ZDHHC4 regulates STAT3 activity in a positive feedback loop. Using a luciferase reporter gene experiment, we found that the transcription of ZDHHC4 was regulated by STAT3. The first mutant fragment (GTTTCAGGAAC), −1785 to −1775 region of the ZDHHC4 promoter is the interaction site (Supplementary Fig. 5A). Moreover, STAT3 silencing and Niclosamide (an inhibitor targeting STAT3) reduced the expression of ZDHHC4 (Supplementary Fig. 5B, C). Niclosamide has been reported to inhibit STAT3 activity [22]. We found that Niclosamide inhibited SF126 cell viability in a concentration-dependent manner (Supplementary Fig. 5D). These results indicated that ZDHHC4-regulated GSK3β palmitoylation activates the EZH2–STAT3 axis (Fig. 2D).

### GSK3β palmitoylation promotes GBM TMZ-resistance

**Fig. 3:**
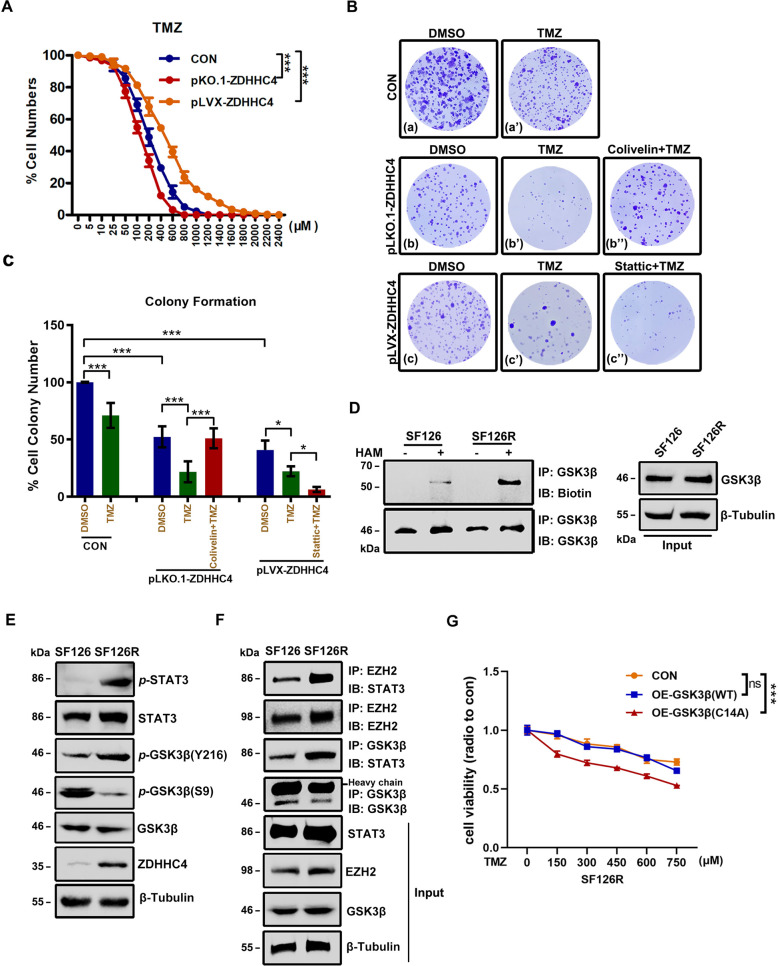
GSK3β palmitoylation regulates GBM TMZ-resistance. **A** Growth curve describing the effect of ZDHHC4 expression on TMZ inhibition in GBM cells. SF126 cells with overexpression or knockout of ZDHHC4 were treated with different concentrations of TMZ. **B** Representative image of colony formation in the SF126 cell lines 14 days after different treatments. For the control group, DMSO and TMZ were used. For the pLKO.1-ZDHHC4 group, DMSO, TMZ, and TMZ + Colivelin were used. For the pLVX-ZDHHC4 group, DMSO, TMZ, and TMZ + Stattic were used. Colonies were stained using crystal violet. **C** The number of clones stained by crystal violet in each dish in Figure B is calculated. Con-DMSO group was set at 100%. **D** ABE analysis of GSK3β palmitoylation in SF126 and SF126R cells. **E** Western blot analysis of GSK3β and STAT3 phosphorylation in SF126 and SF126R cells. **F** Immunoprecipitation analysis of the STAT3 binding capacity of GSK3β and EZH2 in SF126 and SF126R cells. **G** CCK-8 assay was used to detect the effect of GSK3β (C14A) mutant on TMZ killing SF126R cells. Data are shown as means ± SD (*n* = 3). *P*-values were determined by two-tailed Student’s *t*-test. *P < 0.05; **P < 0.01; ***P < 0.001.

Recently, TMZ has been the main treatment for GBM. However, GBM patients are prone to TMZ resistance, resulting in limited therapeutic effects [23]. We found that the expression of ZDHHC4 correlated with the killing ability of TMZ against GBM. When TMZ was used to treat SF126 cells, inhibition of ZDHHC4 improved the killing effect of TMZ on GBM (Fig. 3A, B). Combined with small-molecule drugs in the STAT3 pathway, it was found that after knocking out ZDHHC4, Colivelin, an agonist of STAT3, can inhibit the killing ability of TMZ. Conversely, after overexpression of ZDHHC4, Stattic, an inhibitor of STAT3, enhanced the effect of TMZ (Fig. 3B, C). These results showed that the regulation of TMZ resistance in GBM by ZDHHC4 is mediated by STAT3.

Next, we tested the tolerance of eight glioma cell lines to TMZ. SF126 had poor tolerance to TMZ and no MGMT expression, whereas U118MG and other cells had stronger resistance and higher MGMT expression (Supplementary Fig. 6A-B). The O-6-methylguanine-DNA methyltransferase (MGMT) enzyme removes cytotoxic TMZ alkylation products from DNA and makes cells resistant to TMZ [24]. SF126 and U118MG cells were treated with TMZ into two TMZ-resistant cell lines, SF126R and U118R (R indicates TMZ resistance). SF126R had higher MGMT expression and stronger invasion characteristics than SF126 (Supplementary Fig. 6C, D). In SF126R and U118R cells, GSK3β palmitoylation was enhanced (Fig. 3D; Supplementary Fig. 6E), and the expression of ZDHHC4 was increased, *p-*GSK3β (Y216) and *p-*STAT3 (Y705) were activated, and *p-*GSK3β (S9) was inhibited (Fig. 3E; Supplementary Fig. 6F). The interaction of GSK3β and EZH2 with STAT3 was also enhanced (Fig. 3F). Moreover, the ZDHHC4–GSK3β–STAT3 axis in two TMZ-resistant cell lines was consistently activated, which may indicate that palmitoylation of GSK3β regulates the TMZ resistance of GBM in an additional way. TMZ-resistant GBM cells stably expressing GSK3β (C14A) were more sensitive to TMZ killing than those with wild-type GSK3β protein (Fig. 3G; Supplementary Fig. 7A). These data proved that GSK3β palmitoylation contributes to TMZ resistance in GBM.

### GSK3β palmitoylation promotes GBM TMZ-resistance by regulating the tumorigenicity of GSCs

**Fig. 4:**
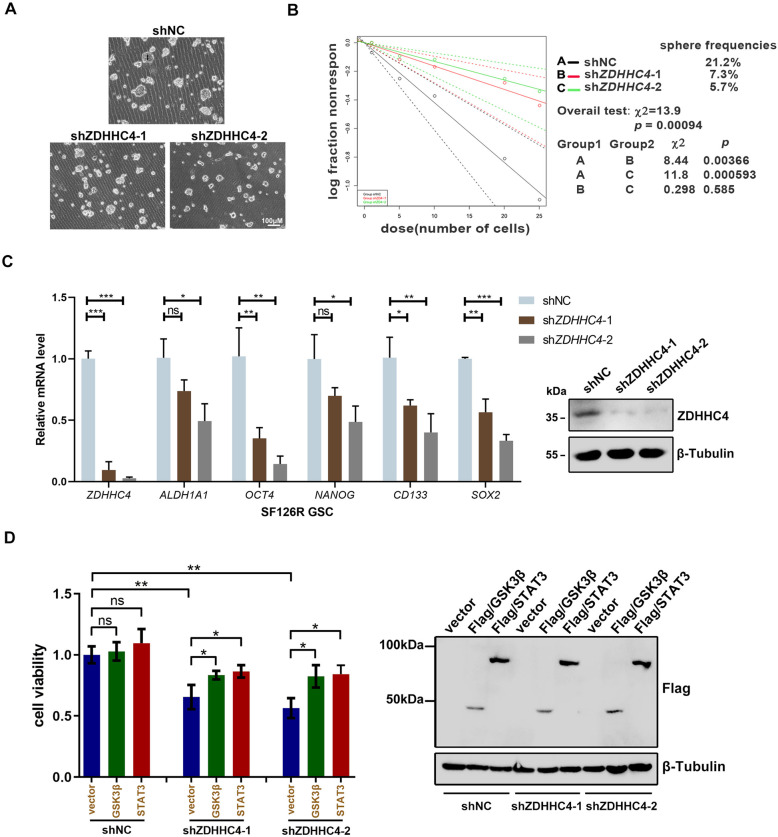
GSK3β palmitoylation contributes to the maintenance of stemness of TMZ-resistant GBM cells. **A** Representative images of GSCs induced from SF126R with ZDHHC4 knockdown. **B** Limiting dilution assay analysis (ELDA) of GSCs showed the frequencies of neurosphere formation. The significance of the difference between the indicated groups was determined by the χ^2^ test (*n* = 3 independent experiments). **C** Left, Real-time PCR analysis shows mRNA levels of STAT3 target stem-cell markers in ZDHHC4-knockdown SF126R GSCs. Control-shRNA cells were set to 1; right, Western blot verifies the effect of ZDHHC4 knockdown. **D** Left, SF126R cells with stable ZDHHC4 knockdown are supplemented with GSK3β or STAT3 for 24 h. Cell viability is measured by CCK-8. The cells stably expressing shNC transfected with empty vector were set to 1; Right, Expression verification of GSK3β and STAT3. Data are shown as means ± SD (*n* = 3). *P*-values were determined by two-tailed Student’s *t*-test. *P < 0.05; **P < 0.01; ***P < 0.001.

Next, we explored how GSK3β palmitoylation promoted TMZ resistance. Due to the tumorigenic and highly invasive ability of GSCs, GBM shows high resistance to radiotherapy and chemotherapy [4]. We first examined the stem-cell pelletizing ability of the four types of GBM cells. Tumor neurospheres induced by SF126R and U118R cell lines were significantly larger than those induced by SF126 and U118MG cells (Supplementary Fig. 7B). Using quantitative real-time PCR (RT-PCR) we observed that mRNA expression of GSC markers (*OCT4, CD133,* and *SOX2*) in neurospheres of SF126R was significantly higher than that of SF126 (Supplementary Fig. 7C). SF126R and U118R cells were used to construct cell lines with stable knockdown of ZDHHC4. Silencing ZDHHC4 reduced the spheroidizing ability of SF126R cells (Fig. 4A). In the extreme limiting dilution assay (ELDA), the depletion of ZDHHC4 obviously inhibited the formation of tumor spheres in SF126R cells as evidenced by the decrease in the number, size, and frequency of stem cells (Fig. 4B). The expression of several stem-cell markers (*ALDH1A1, OCT4, NANOG, CD133,* and *SOX2*) decreased with the downregulation of ZDHHC4 (Fig. 4C), which was verified in U118R and TMZ-intolerant SF126 cells (Supplementary Fig. 7D-E).

In addition, the silencing of ZDHHC4 inhibited the incorporation of EdU in SF126R and U118R cells to reduce proliferation and promote cell apoptosis (Supplementary Fig. 8A-B). However, when ZDHHC4 was knocked down, supplementation with GSK3β and STAT3 rescued the viability of TMZ-resistant cells (Fig. 4D). These results suggest that ZDHHC4 enhances TMZ resistance in GBM cells by promoting stemness and proliferation while inhibiting apoptosis.

### Knockdown of ZDHHC4 restores TMZ sensitivity in vivo

**Fig. 5:**
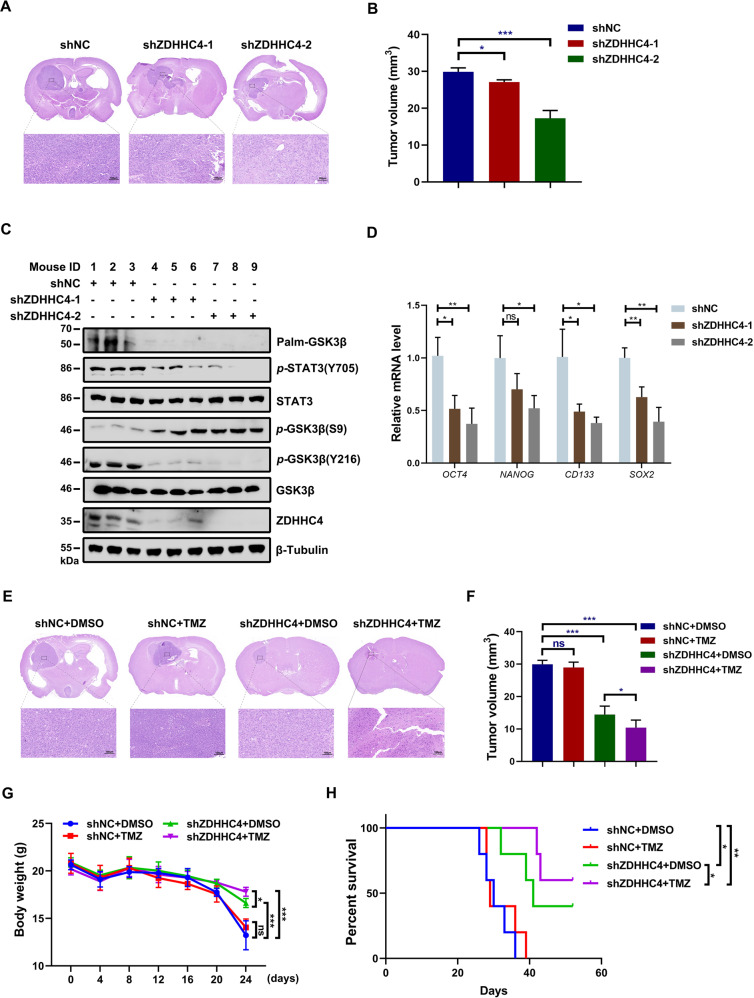
GBM with ZDHHC4 knockdown in vivo was more sensitive to TMZ treatment. **A** U118R cells (shNC, shZDHHC4-1, and shZDHHC4-2, *n* = 5/group) (5 × 10^5^ cells/mouse) were injected into nude mice. Mice were sacrificed 45 days later. H&E staining demonstrated typical tumor xenografts. **B** Intracranial tumor volumes in panel **A** were calculated (mean ± SD, *n* = 5 for each group, two-tailed Student’s *t*-test). **C** GSK3β palmitoylation and GSK3β-STAT3 pathway activity in tumors were detected by western blot. **D** The mRNA levels of GSC markers (*OCT4, NANOG, CD133,* and *SOX2*) in tumor tissues at the end of the experiment were analyzed by RT-PCR. The folding changes were normalized to shNC (mean ± SD, *n* = 5 for each group, two-tailed Student’s *t*-test). **E** U118R cells (shNC and shZDHHC4-2 each in two groups, *n* = 5/group) were injected into nude mice. Three days after cell injection, mice were intraperitoneally injected with TMZ (25 mg kg^−1^ d^−1^) every other day for 30 days. Mice were sacrificed humanely 45 days later. H&E staining demonstrated typical tumor xenografts. **F** Intracranial tumor volumes in (**E**) were calculated (mean ± SD, *n* = 5 for each group, two-tailed Student’s *t*-test). **G** The mice were weighed every 4 days (mean ± SD, *n* = 5 for each group, two-tailed Student’s *t*-test). **H** Kaplan–Meier survival curves were used to define the overall survival of intracranial tumor-bearing mice.

To clarify the role of GSK3β palmitoylation in TMZ resistance in GBM in vivo, we established an orthotopic model of mice carrying U118R xenografts. We first found that ZDHHC4 silencing significantly reduced tumorigenesis in vivo (Fig. 5A, B). As ZDHHC4 decreased, *p-*GSK3β (Y216) and *p-*STAT3 (Y705) decreased in tissues and cells of the xenograft model, while the expression of *p-*GSK3β (S9) increased (Fig. 5C; Supplementary Fig. 9A). Palmitoylation of GSK3β was also significantly inhibited (Fig. 5C). In addition, the mRNA levels of *OCT4, NANOG, CD133*, and *SOX2* also decreased with ZDHHC4 silencing (Fig. 5D). TMZ treatment alone did not reduce the volume of the tumor, and knockdown of ZDHHC4 effectively restored the sensitivity of the tumor tissue to TMZ (Fig. 5E, F). The mice treated with the combination therapy showed significantly smaller tumor volumes (Fig. 5F), more stable body weight (Fig. 5G), and increased longevity (Fig. 5H) than the other groups. These data suggest that targeting ZDHHC4 could effectively improve sensitivity to TMZ treatment.

### ZDHHC4 is an unfavorable prognostic factor for GBM patients

**Fig. 6:**
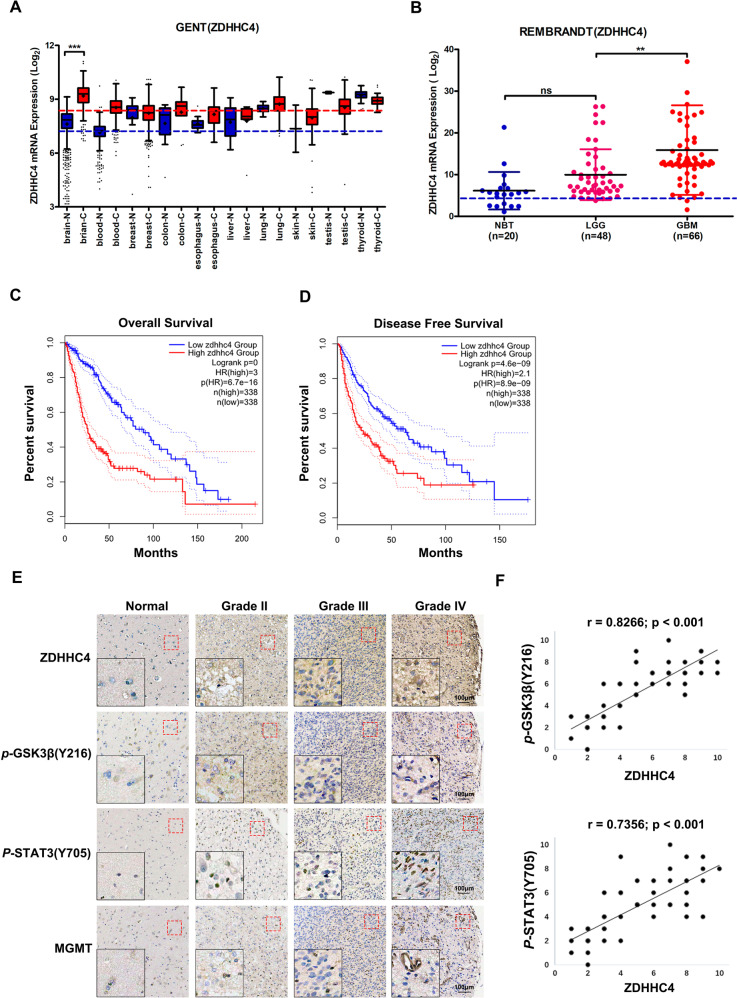
The ZDHHC4–GSK3β–STAT3 axis was correlated with the malignant degree of human glioma. **A** Differences in ZDHHC4 expression in tumors and normal tissues of various organs obtained from the GENT database. **B** Relationship of ZDHHC4 to glioma grade from the REMBRANDT database. **C**, **D** Diagram of ZDHHC4 expression with overall survival and disease-free survival in glioma patients obtained from TCGA database. **E** Immunohistochemical staining showed that ZDHHC4 expression was correlated with *p-*GSK3β (Y216), *p-*STAT3 (Y705), and MGMT; 125 biologically independent samples were analyzed. The red dotted line marks the area corresponding to the high magnification image. **F** Statistical analysis of the Pearson correlation between the immunohistochemical staining score of ZDHHC4 expression and *p-*GSK3β (Y216) or *p-*STAT3 (Y705) expression.

To explore the potential application of GSK3β palmitoylation in the clinical treatment of GBM, we searched the GENT, REMBRANDT, and The Cancer Genome Atlas (TCGA) databases. Compared with normal brain tissue, the expression level of ZDHHC4 was higher in low-grade glioma (LGG) and GBM, especially GBM (Fig. 6A, B; Supplementary Fig. 9B). In terms of glioma patient prognosis, higher levels of ZDHHC4 represented shorter overall survival and disease-free survival, which were obtained from TCGA, Chinese Glioma Genome Atlas (CGGA), and REMBRANDT (Fig. 6C, D; Supplementary Fig. 9C D).

Immunohistochemical staining results of 125 brain tissue samples showed that the expression trends of ZDHHC4, *p-*GSK3β (Y216), and *p-*STAT3 (Y705) were consistent and positively correlated with the pathological grade of glioma (Fig. 6E; Supplementary Table S7-9). Quantification of staining on a scale of 0–10 points shows that these correlations were significant (Fig. 6F). Thus, these results further revealed that the activity of ZDHHC4 was positively associated with the activation of GSK3β and STAT3 in human glioblastomas.

**Fig. 7:**
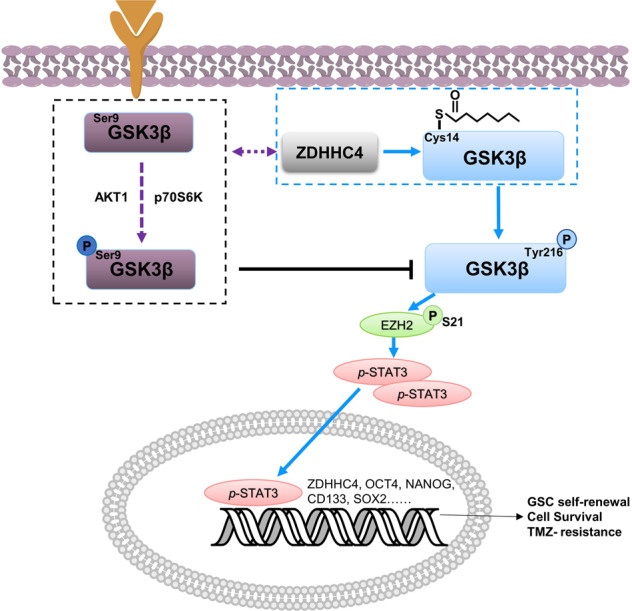
Schematic showing that ZDHHC4-mediated GSK3β palmitoylation promotes GBM TMZ-resistance. ZDHHC4 palmitoylates GSK3β and competitively binds GSK3β with AKT and p70S6K kinases, which inhibits GSK3β Ser9 phosphorylation and enhances GSK3β Tyr216 phosphorylation. Activated GSK3β enhances GSC self-renewal, GBM TMZ-resistance, and tumorigenesis by promoting the EZH2/STAT3 axis.

Taken together, GSK3β palmitoylation medicated by ZDHHC4 could decrease *p-*Ser9 and increase *p-*Tyr216 for GSK3β, leading to activate the EZH2/STAT3 axis to promote GSC tumorigenicity and ultimately enhance TMZ resistance in GBM (Fig. 7).

## Discussion

Although it plays an important role in tumors, the mechanism and regulatory network of GSK3β in gliomas have not been elucidated. Our experiment revealed a novel post-translational modification of GSK3β, mediated by the palmityl transferase ZDHHC4. Palmitoylated GSK3β promotes GBM stemness by activating STAT3 phosphorylation, thereby making GBM resistant to TMZ.

The regulation patterns of GSK3β among tumor types are different or even completely opposite [25]. On the one hand, GSK3β has been recognized as a tumor suppressor because it downregulates many proto-oncoproteins and cell cycle checkpoint proteins, thereby inhibiting cell proliferation [9, 26, 27]. On the other hand, GSK3β is also a tumor promoter. GSK3β is overexpressed in various tumor types [9]. In GBM, the consensus view is that GSK3β promotes tumorigenesis [28–30]. Our study further found that GSK3β mediated the development of TMZ resistance in GBM.

The function of GSK3β depends mainly on its phosphorylation [11]. We found that palmityl transferase ZDHHC4 competitively bound GSK3β with AKT and p70S6K to attenuate Ser9 phosphorylation. Protein palmitoylation is the most important lipid modification. It regulates protein activity, subcellular localization, and signal transmission [16]. The effect of palmitoylation on tumorigenesis is mostly through the modification of key molecules (EGFR, RAS, etc.) of tumor proliferation, death resistance, and tumor metastasis, causing abnormalities in signaling pathways, metabolism, and gene expression regulation [31, 32]. However, our results showed that the change in GSK3β palmitoylation did not affect the level of β-catenin. This is probably because only a few GSK3β (≈10%) are present in the β-catenin destroying complex in cells or the GSK3β in the complex is structurally shielded from contact with other intracellular proteins [7].

Previous research has reported that GSK3β regulates the phosphorylation activity of STAT3, but the specific regulatory mechanism has not been identified [33]. Our work is the first to demonstrate that palm-GSK3β phosphorylates EZH2 (S21), which in turn regulates STAT3 methylation and phosphorylation. However, how GSK3β binds to EZH2 needs to be further explored. The EZH2–STAT3 axis is one of the key regulatory pathways of GSC self-renewal [15]. GSK3β has also been shown to help maintain GSC self-renewal in several studies [25, 30, 34]. The combination of these factors has enhanced our understanding of the central role of GSK3β in GBM development. One of the major manifestations of refractory cancer is tumor cell tolerance to chemotherapeutic drugs. The existence of GSCs and the elevated expression of MGMT are two major reasons for TMZ resistance in GBM [35]. Our study showed that GSC self-renewal of SF126R and U118R cells was enhanced and that ZDHHC4–Palm/GSK3β–STAT3 signaling was continuously activated under continuous stimulation with TMZ, regardless of whether the expression of MGMT was changed. This shows that the enhancement of stemness of GBM cells is the main reason for the regulation of TMZ resistance by GSK3β palmitoylation. In addition, the positive feedback loop formed by STAT3 regulation of ZDHHC4 expression also provides a theoretical basis for clinical refractory GBM.

In conclusion, we showed that ZDHHC4-mediated palmitoylation of GSK3β is critical for its kinase activity. Depending on EZH2–STAT3 activity, GSK3β palmitoylation promotes GSC self-renewal and TMZ resistance in GBM (Fig. 7). Taken together, this study reveals the mechanism of GSK3β palmitoylation in GBM occurrence and further emphasizes that GSK3β is a promising therapeutic target for GBM.

## Supplementary information

Supplementary Information

Authorship Change Agreement

## Data Availability

All data generated or analyzed during this study are included within this article.
