# Supplementary material for: GSK3β palmitoylation mediated by ZDHHC4 promotes tumorigenicity of glioblastoma stem cells in temozolomide-resistant glioblastoma through the EZH2–STAT3 axis
Source: Oncogenesis. 2022 May 23;11(1):28. doi: 10.1038/s41389-022-00402-w (PMC9126914; doi:10.1038/s41389-022-00402-w)
Supplement: Supplementary file 1 — Supplementary Information [file 41389_2022_402_MOESM1_ESM.docx]

SUPPLEMENTARY INFORMATION

**GSK3β palmitoylation mediated by ZDHHC4 promotes tumorigenicity of glioblastoma stem cells in temozolomide****-resistant glioblastoma through the EZH2-STAT3 axis**

**Authors and affiliations**

Chenggang Zhao PhD,^1,2,3^ Huihan Yu MD,^1,3^ Xiaoqing Fan PhD,^1,2,4^ Wanxiang Niu PhD,^1,2^  Junqi Fan MD,^1,2^ Suling Sun MD,^1,2^ Meiting Gong MD,^1,3^ Bing Zhao PhD,^5, *^ Zhiyou Fang PhD,^1,3,*^ Xueran Chen PhD,^1,3,*^

^1^ Anhui Province Key Laboratory of Medical Physics and Technology; Institute of Health and Medical Technology, Hefei Institutes of Physical Science, Chinese Academy of Sciences, No. 350, Shushan Hu Road, Hefei, Anhui, 230031, China; MOE Key Laboratory for Membraneless Organelles and Cellular Dynamics, University of Science & Technology of China, No. 96, Jin Zhai Road, Hefei, Anhui, 230027, China

^2^ Science Island Branch, Graduate School of University of Science and Technology of China, No. 96, Jin Zhai Road, Hefei, Anhui, 230031, China

^3^ Department of Laboratory Medicine, Hefei Cancer Hospital, Chinese Academy of Sciences, No. 350, Shushan Hu Road, Hefei, Anhui, 230031, China

^4^ Department of Anesthesiology, The First Affiliated Hospital of USTC, Division of Life Sciences and Medicine, University of Science and Technology of China (USTC), No. 17, Lu Jiang Road, Hefei, Anhui, 230001, China

^5^ Department of Neurosurgery, The Second Affiliated Hospital of Anhui Medical University, No. 678, Fu Rong Road, Hefei, Anhui, 230601, China.

**Corresponding author**

**^*^**Correspondence should be directed to Pro. Bing Zhao (aydzhb@126.com), Pro. Zhiyou Fang [(zyfang@cmpt.ac.cn),](mailto:(zyfang@cmpt.ac.cn),) and Dr. Xueran Chen (xueranchen@cmpt.ac.cn).

**This PDF file includes:**

1. Materials and Methods
2. Figures. S1-S9
3. Tables. S1-S9

**SUPPLEMENTARY MATERIALS AND METHODS**

**Proximity Ligation Assay (PLA)**

Proximity ligation assays (PLA) was performed with the mouse/rabbit red starter Duolink kit (Sigma-Aldrich) as per manufacturer's instructions. Briefly, SF126 cells were fixed with 4% PFA for 20 min. and permeabilized with 0.5% Triton PBS for 10 min. Cells were incubated in the blocking buffer at 37°C for 1 hr in a humidified chamber. Next, cells were incubated with the mouse anti‐PKA Antibody (1:100, Santa Cruz), anti-p90RSK Antibody (1:50, Santa Cruz), anti-p70S6K Antibody (1:100, Santa Cruz), or anti-AKT1 Antibody (1:200, Santa Cruz) and the rabbit anti‐ZDHHC4 (1:500, Abcam) at 4°C overnight. After wash, cells were then incubated with the PLA probes at 37°C for 1 hr, and then ligation was performed at 37°C for 1 hr in a humidified chamber. Cells were then incubated with the amplification mix at 37°C for 2 hrs in a darkened humidified chamber. Cells were then mounted on coverslips using the mounting media supplied with the kit and imagined using an inverted microscope (Leica Inverted Microscope).

**Luciferase assay**

pGL3 Vector was purchased from Promega (USA). The pcDNA3.1 vector and pcDNA3.1-Flag-STAT3 were co-transfected into 293T cells with pGL3-ZDHHC4 plasmids containing different regions. After 48 h, the Firefly Luciferase Reporter Gene Assay Kit (Beyotime, Shanghai, China) was used to determine the luciferase activity.

**Screening of TMZ resistant cell lines**

SF126 and U118MG cells were treated with TMZ at initial concentrations of 300 µM and 400 µM for one week, respectively. The TMZ concentration was increased by 50 µM every two cell passages until cell death ceased at 800 µM.

**Cell invasion assay, cell apoptosis assay and cell proliferation assay**

For the cell invasion assay, SF126 and SF126R cells were added to Transwell nested chambers (Corning, USA) combined with Matrigel in a 24-well plate. After 16 hrs, the transmembrane cells outside the chamber were stained with crystal violet. Five field counts were randomly taken, and the experiment was repeated three times.

Cell apoptosis assays were performed using an Annexin V-FITC Apoptosis Detection Kit (Beyotime, Shanghai, China) following the manufacturer's instructions for experimental procedures.

A Cell-Light EdU Apollo488 In Vitro Kit (RIBOBIO, Guangzhou, China) was used to detect cell proliferation. The experimental procedures were performed according to the manufacturer's instructions and repeated twice.

**SUPPLEMENTARY FIGURES**


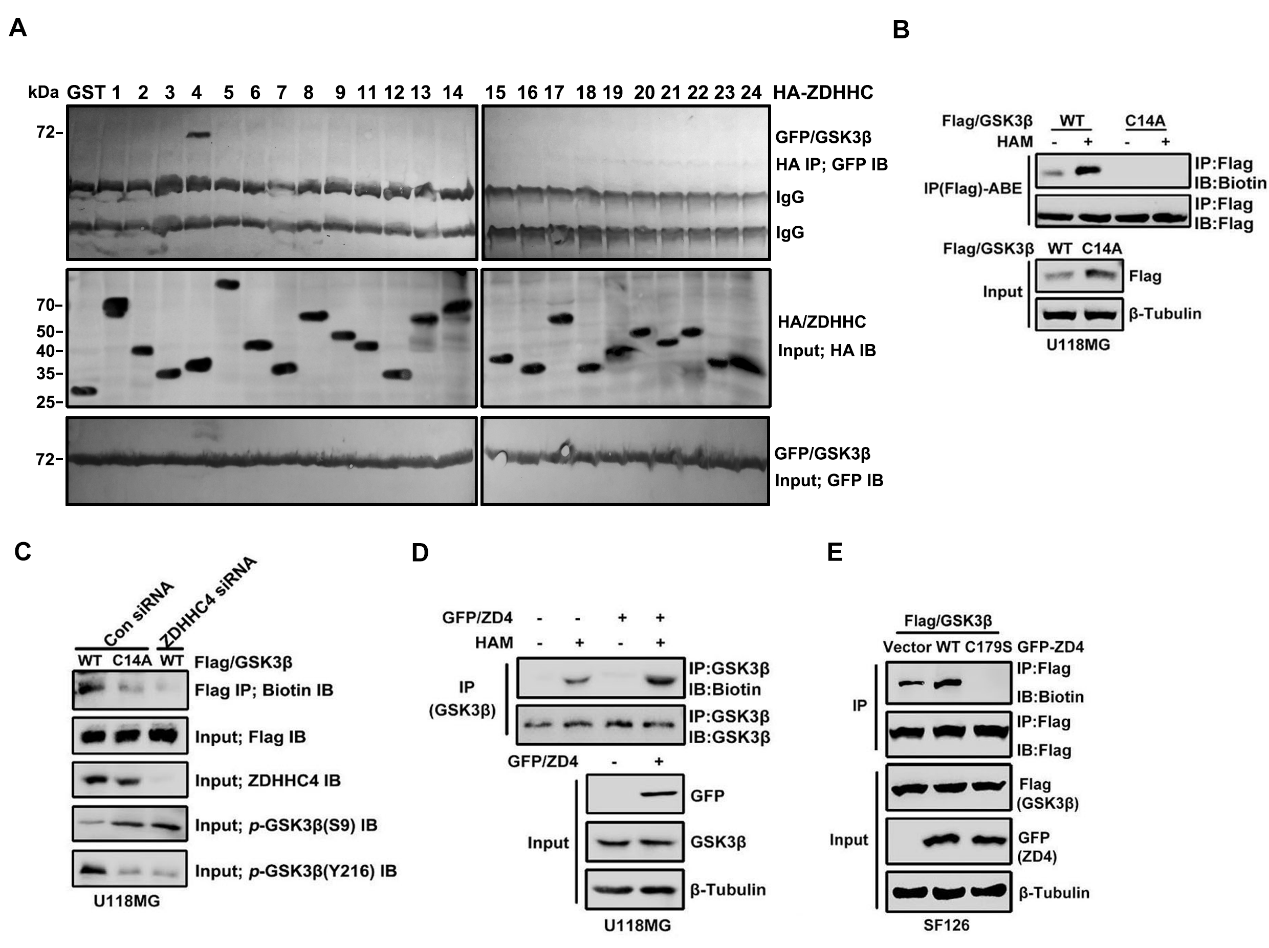


**Supplementary Fig S1. ZDHHC4 regulates GSK3β palmitoylation.**

**A,** The interaction between 23 HA-ZDHHCs and GFP-GSK3β was detected by immunoprecipitation in 293T cells. **B,** ABE analyzed the effect of C14A mutant on GSK3β palmitoylation in U118MG cell line. **C,** U118MG cells were transfected, and the experiment was divided into three groups: wild-type Flag-GSK3β, C14A mutant GSK3β and wild-type Flag-GSK3β were simultaneously knocked down ZDHHC4 by siRNA. ABE analysis and phosphorylation of GSK3β were performed in the three groups. **D,** U118MG cells were transfected GFP-ZDHHC4 plasmid for 24 hrs, GSK3β palmitoylation level was detected by ABE, followed by immunoblotting. **E,** Effects of ZDHHC4 (C179S) mutant on palmitoylation of GSK3β.


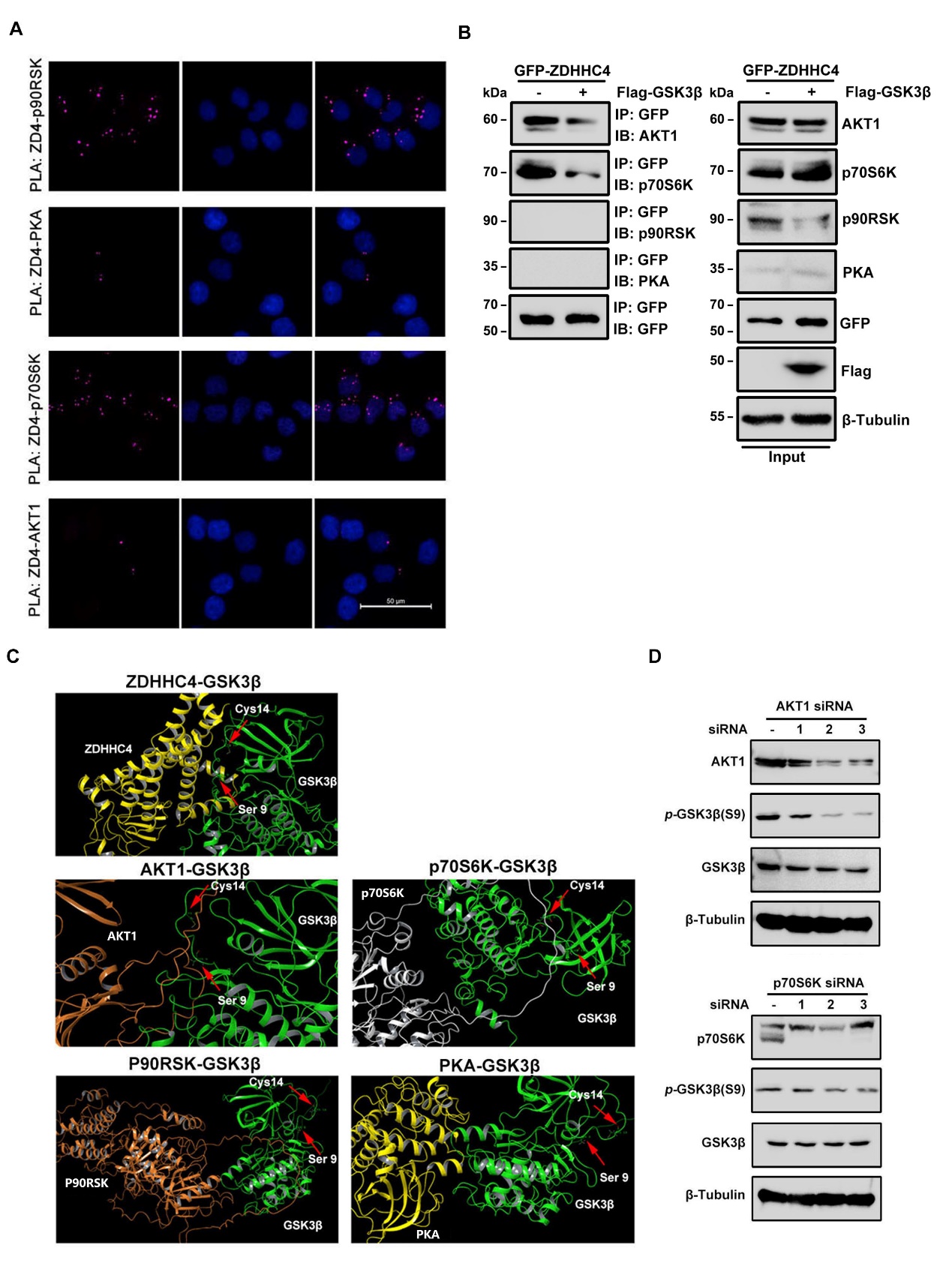


**Supplementary Fig S2. ZDHHC4 competes with AKT1 and p70S6K for binding to GSK3β protein.**

**A,** Proximity ligation assays (PLA) were used to determine the binding capacity of four kinases to GSK3β. **B,** GFP-ZDHHC4 was transfected into SF126 cells with/without stable expression of Flag-GSK3β. Immunoprecipitation assays showed GFP-ZDHHC4 interactions with AKT, p70S6K, PKA, and p90RSK. **C,** The molecular simulation software Schrodinger Suites 2020.3 was used to predict the docking between the kinases and GSK3β protein. **D,** Phosphorylation of GSK3β Ser9 was detected by siRNA knockdown of AKT and p70S6K.


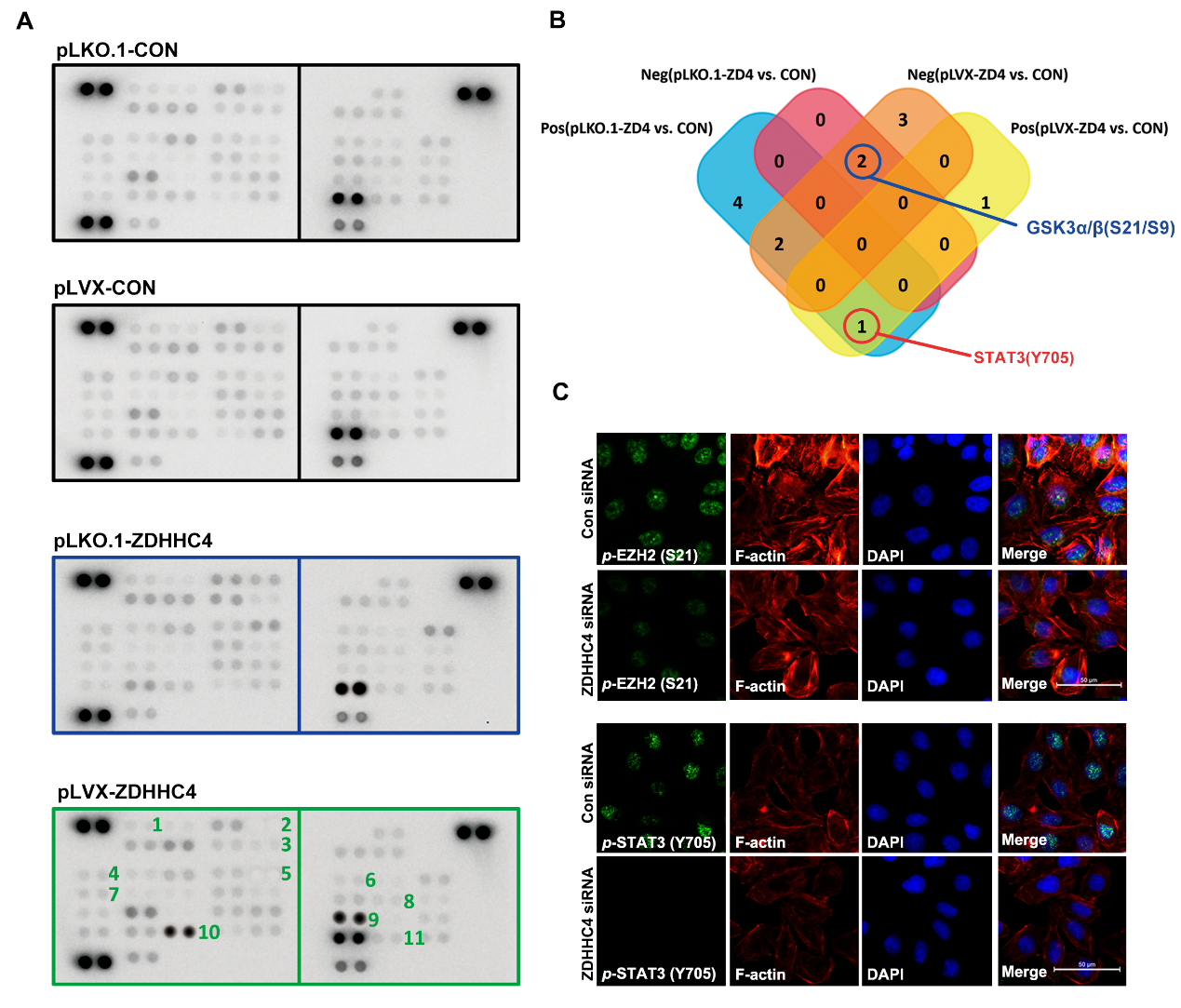


**Supplementary Fig S3. *p-*STAT3 (Y705) was positively correlated with the expression of ZDHHC4.**

**A,** Protein microarray screening for protein signals associated with ZDHHC4 expression. **B,** Venn diagram showing the protein set upregulated/downregulated by ZDHHC4. **C,** Effect of ZDHHC4 siRNA transfection on the expression of *p-*EZH2 (S21) and *p-*STAT3 (Y705) in SF126 cells by immunofluorescence assay.


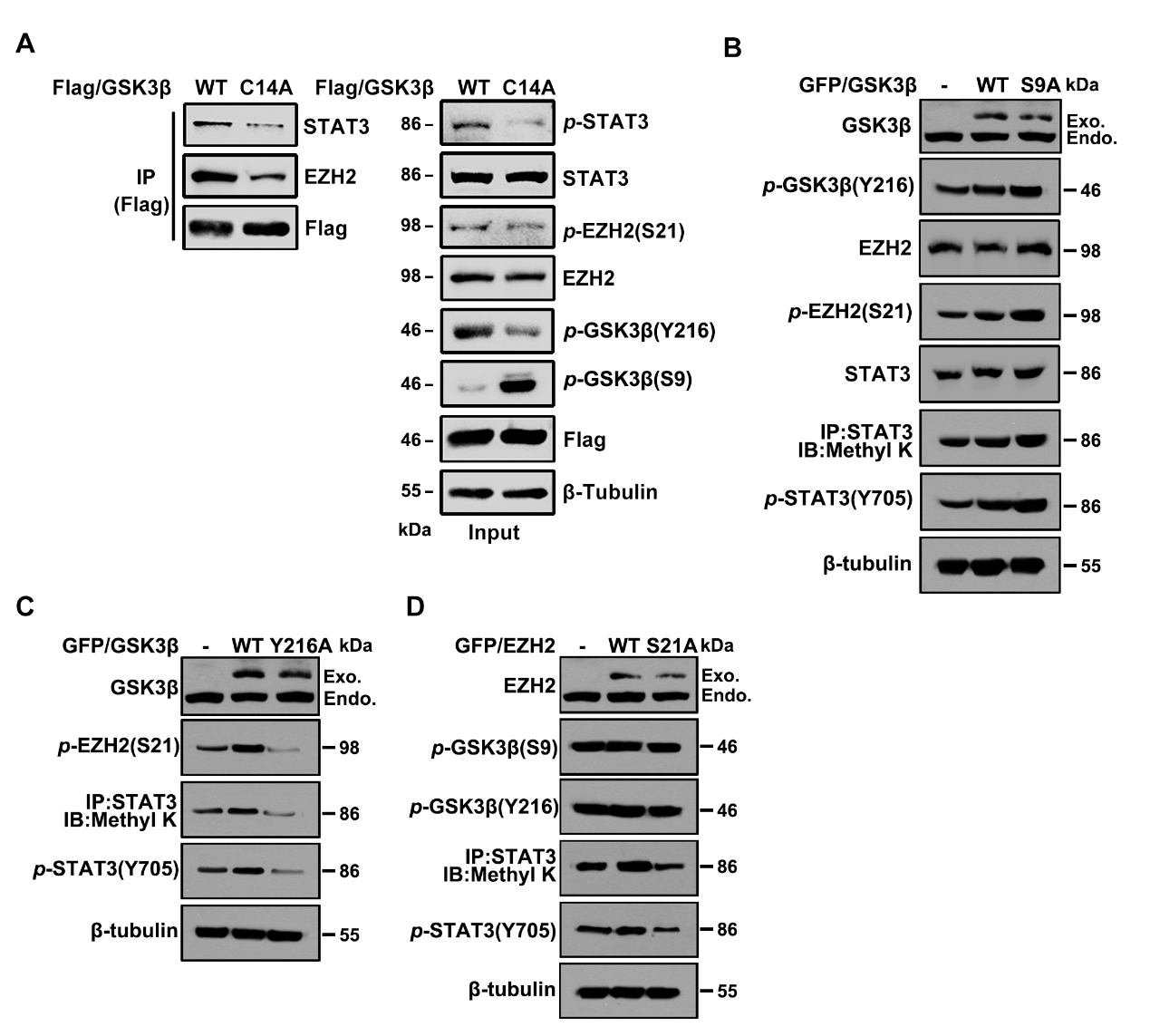


**Supplementary Fig S4. Mutated GSK3β and EZH2 regulates STAT3 activity.**

**A,** Flag-GSK3β (WT) and Flag-GSK3β (C14A) mutants were transfected to detect the binding ability of GSK3β to EZH2 and STAT3 (left) and protein activity (right). **B-D,** Western blot analysis showing the effects of WT and mutants of GSK3β or EZH2 on STAT3 signaling in SF126 cells.


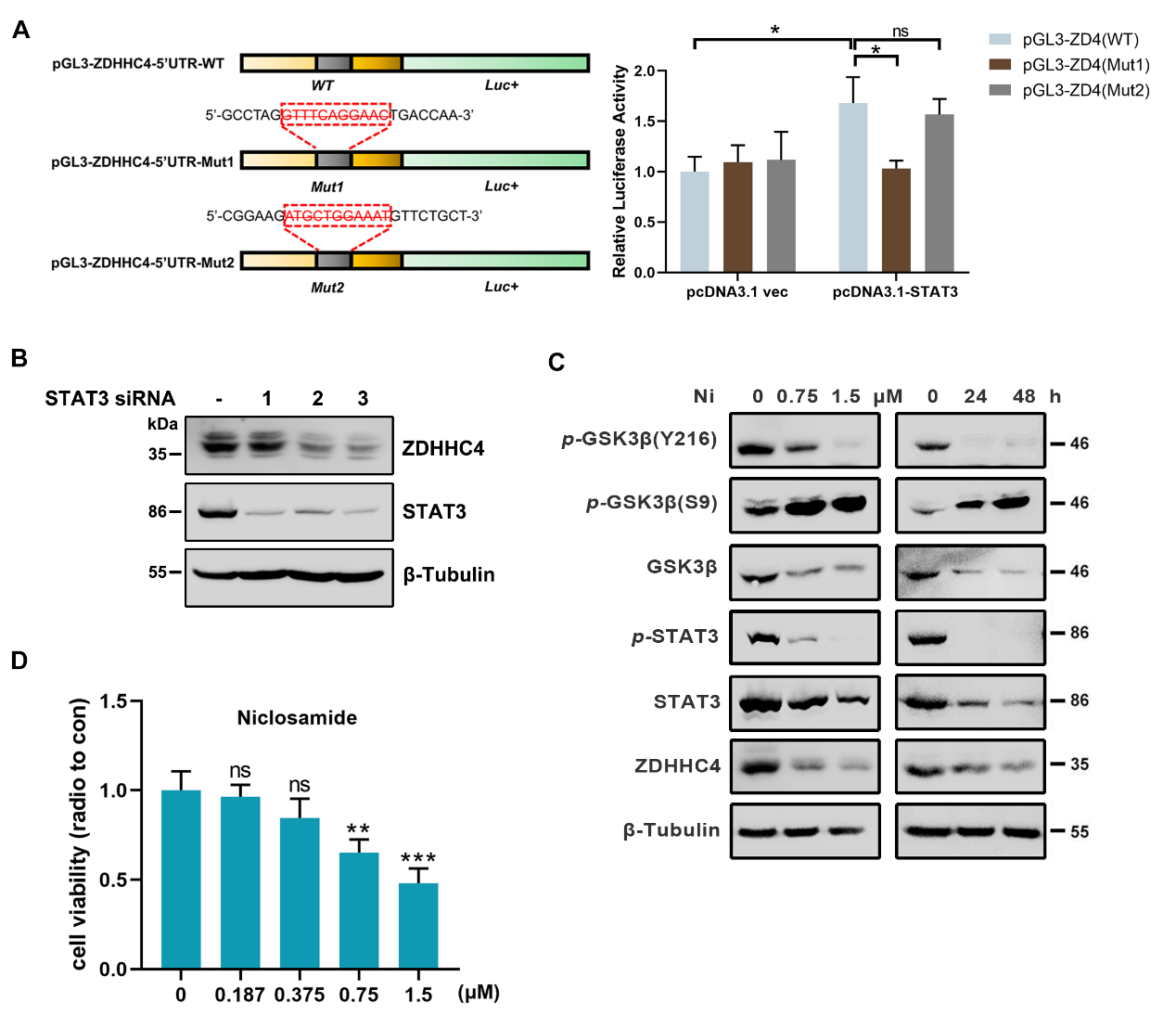


**Supplementary Fig S5. The transcription factor STAT3 promotes the transcriptional expression of ZDHHC4.**

**A,** Luciferase assay showing the binding ability of STAT3 to ZDHHC4 gene promoter region. **B,** Western blot analysis of SF126 cells that were cultured for 48 hours after treatment with control or STAT3 siRNA. **C,** SF126 cells were treated with Niclosamide at an indicated concentration for 48h (left image) or Niclosamide at a final concentration of 1.5 µM for a specified time (right image). Western blot analysis of cell lysate was performed with indicated antibodies. **D,** CCK-8 detected the cell viability of SF126 treated with different concentrations of Niclosamide (0 µM set to 1) (mean±s.d., n=5 holes for each group, one-way ANOVA test).


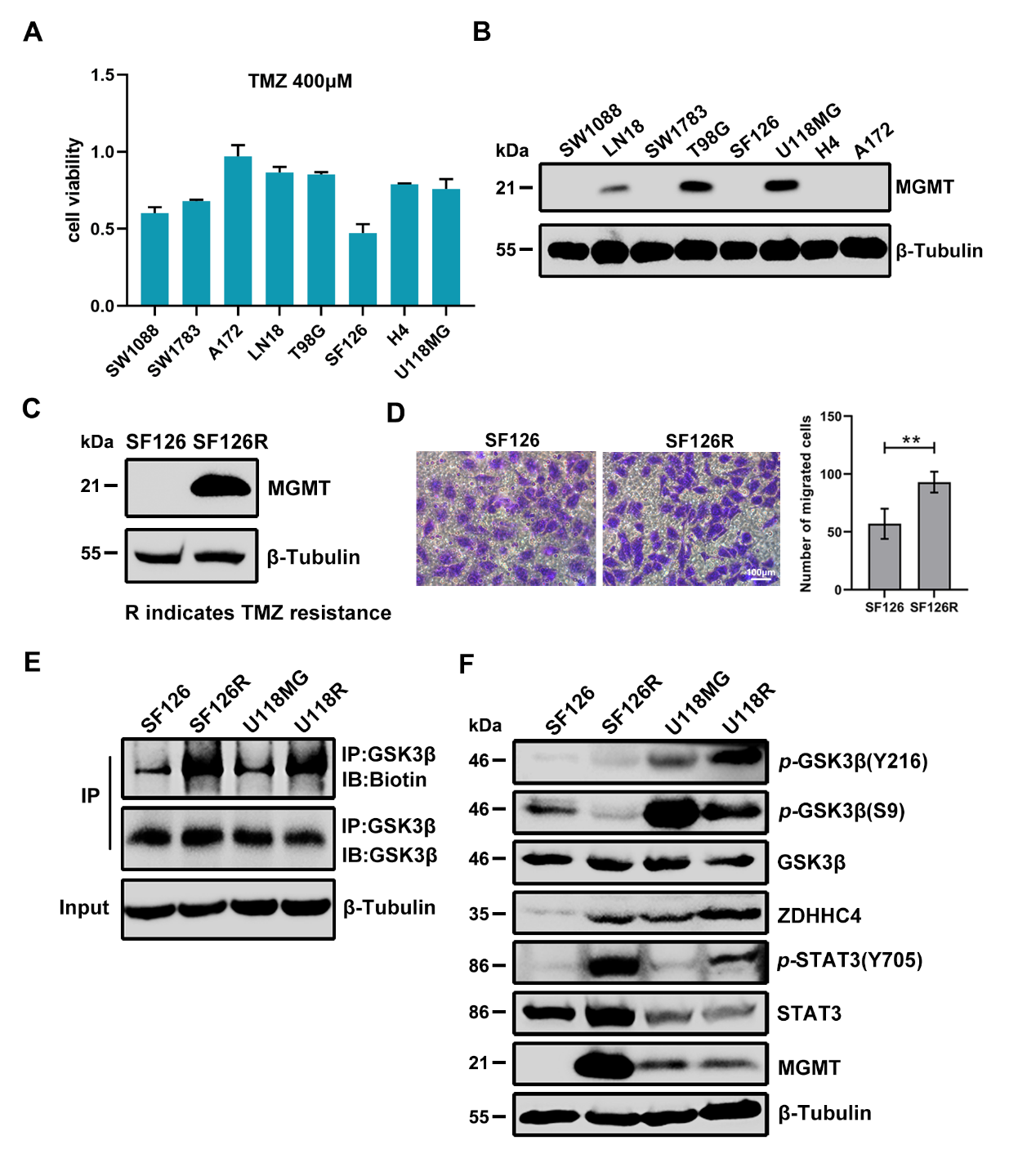


**Supplementary Fig S6. Screening of TMZ resistant GBM cell line.**

**A,** CCK-8 assay was used to detect TMZ tolerance of 8 glioma cell lines. **B,** Western blot analysis showing the protein expression level of MGMT in 8 glioma cell lines. **C,** Western blot analysis of MGMT expression in SF126 and SF126R cell lines. **D,** Transwell assay was performed on the migration of SF126 and SF126R cells. **E,** The intracellular GSK3β was biotinylated by ABE method, and the palmitoylated GSK3β levels in the four cell lines were detected by western blot. **F,** Western blot analysis of signaling pathways in four cell lines.


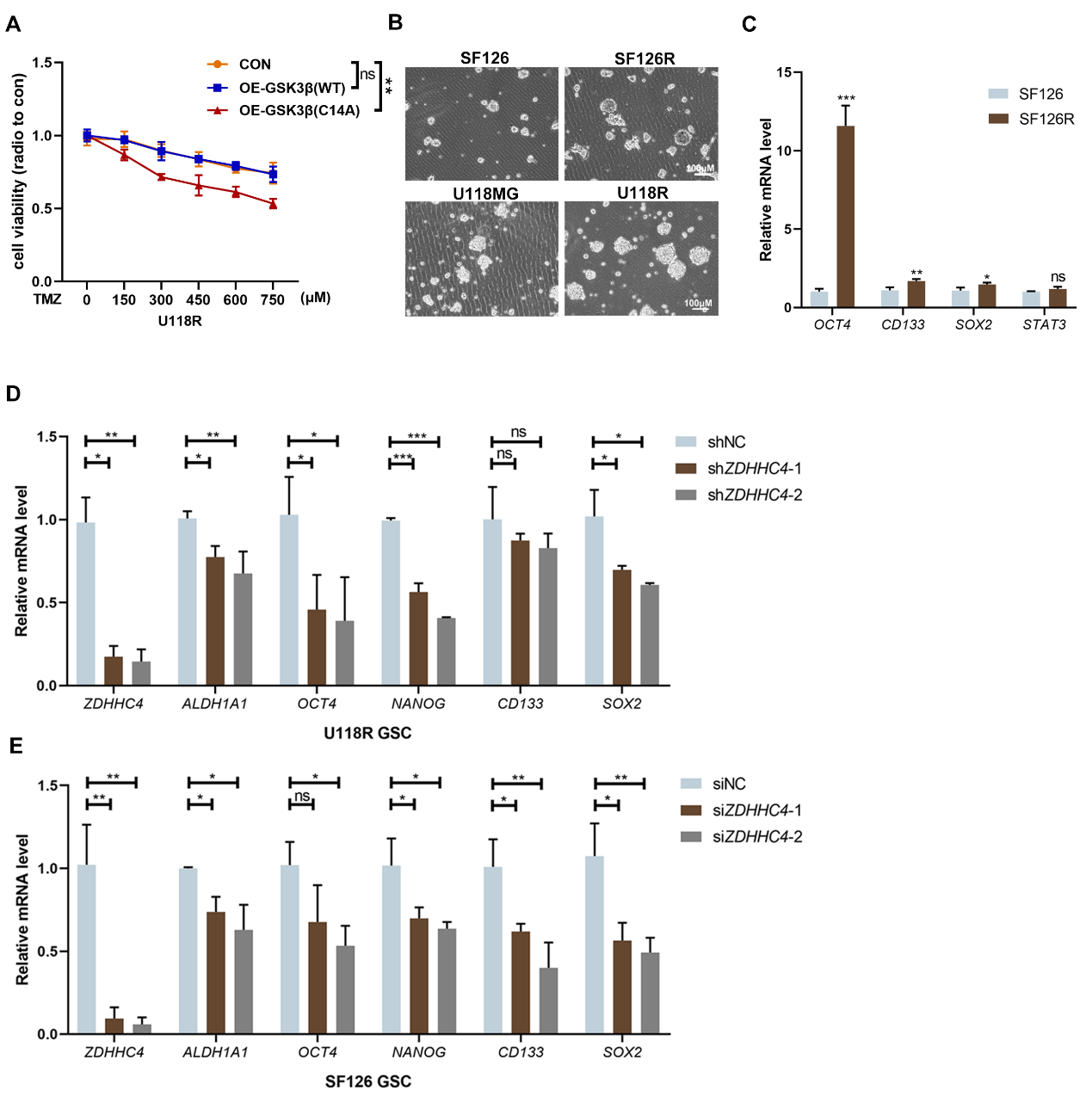


**Supplementary Fig S7. GSK3β palmitoylation inhibits TMZ-resistant cells stemness.**

**A,** CCK-8 assay was used to detect the effect of GSK3β (C14A) mutant on TMZ killing U118R cells. Data are means ± SD (n = 3). *P* values were determined by two-tailed Student’s t test. * *P* < 0.05; ** *P* < 0.01; *** *P* < 0.001. **B,** Representative images of GSCs induced from SF126, SF126R, U118, and U118R. Bar represent 100 microns. **C,** Real-time PCR analysis to determine the mRNA levels of STAT3 target stem-cell markers in SF126 and SF126R GSCs. The mRNA levels of *OCT4, CD133, SOX2, and STAT3* in SF126R cells were compared with those in SF126 cells (set to 1).

**D-E,** Real-time PCR analysis showing mRNA levels of STAT3 target stem-cell markers in ZDHHC4-knockdown U118R and SF126 GSCs. Control-shRNA cells were set to 1.


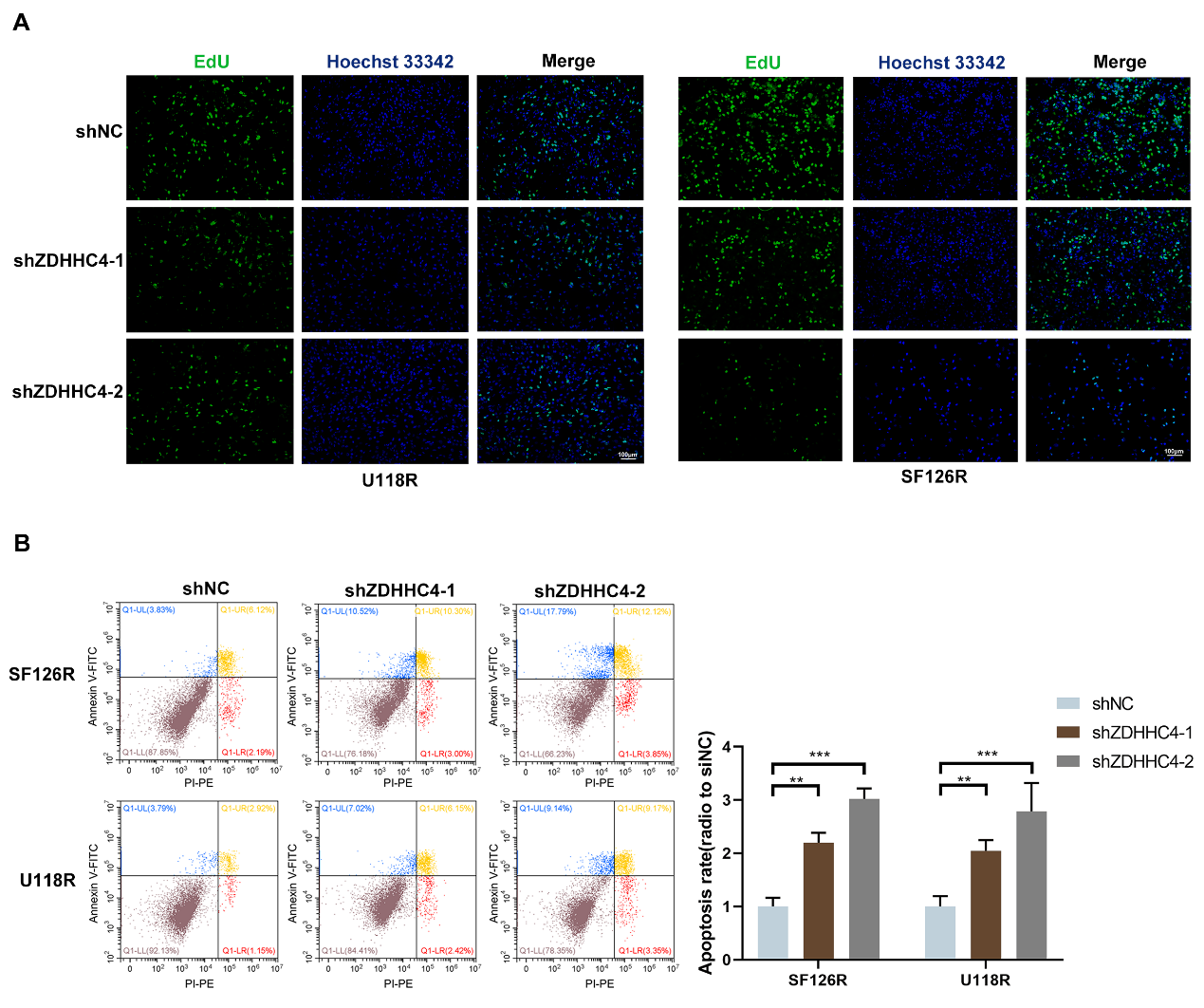


**Supplementary Fig S8. ZDHHC4 silencing inhibits proliferation and promotes apoptosis of TMZ resistant cells.**

**A,** The effect of ZDHHC4 silencing on SF126R and U118R cell proliferation was analyzed by EdU experiment. **B,** Left, The effect of ZDHHC4 silence on SF126R and U118R cell apoptosis was analyzed by PI-Annexin V double staining; Right, Statistical analysis of apoptosis rate. Data are means ± SD (n = 3). *P* values were determined by two-tailed Student’s t test. * *P* < 0.05; ** *P* < 0.01; *** *P* < 0.001.


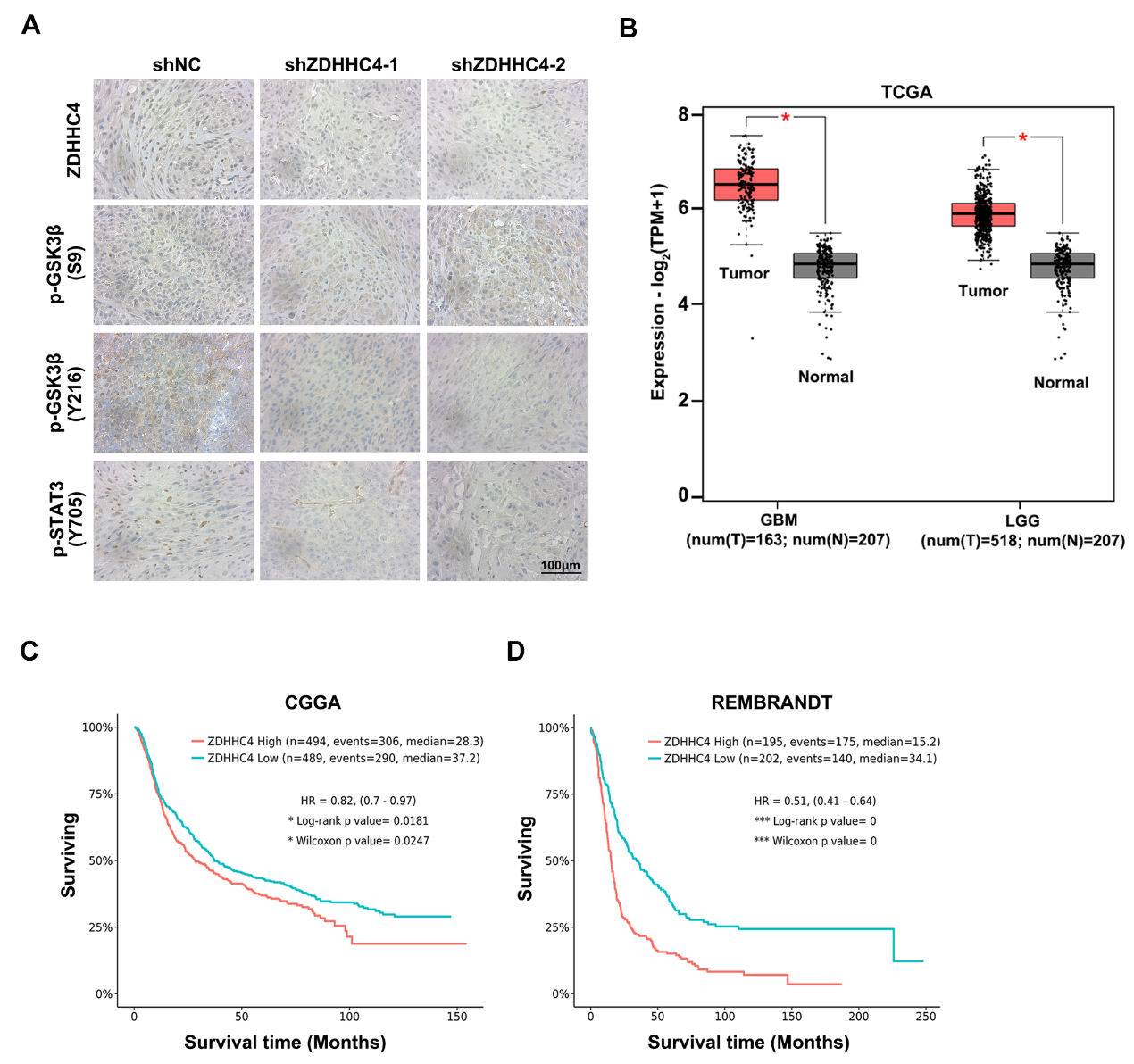


**Supplementary Fig S9. Clinical correlation analysis of ZDHHC4.**

**A,** IHC was used to detect GSK3β-STAT3 pathway activity in tumor tissues. **B,** Analysis of ZDHHC4 expression differences between tumor and normal tissues of GBM and LGG obtained from TCGA database. **C-D,** Correlation analysis of ZDHHC4 expression with glioma survival from CGGA and REMBRANDT databases.

**SUPPLEMENTARY TABLES**

**Supplementary Table S1.**

**Primer sequences of point mutant plasmids.**

| **Gene** | **Sense** | **Antisense** |
| --- | --- | --- |
| GSK3β (C14A) | TCCTTTGCGGAGAGCGCCAAGCCGGTGC | CTGCTGCACCGGCTTGGCGCTCTCCGCA |
| GSK3β (S9A) | CGGCCCAGAACCACCGCCTTTGCGGAGA | GCAGCTCTCCGCAAAGGCGGTGGTTCTG |
| GSK3β (Y216A) | acccaatgtttcgtagctctgttctcgg | tagtaccgagaacagagctacgaaacat |
| EZH2 (S21A) | GGAAGCGTGTAAAAGCAGAGTACATGCGAC | GTCGCATGTACTCTGCTTTTACACGCTTCC |
| ZDHHC4 (C179S) | CGTTTCGACCATCACTCTGTTTGGGTGA | GTTGTTCACCCAAACAGAGTGATGGTCG |

**Supplementary Table S2.**

**Target gene sequence of shZDHHC4.**

| shNC | GATCCGGTTCTCCGAACGTGTCACGTTTTTCAAGAGAACTCTCATGACATGGAAAGGCTTTTTTG |
| --- | --- |
| shZDHHC4-1 | GATCCGCCTTTCCATGTCATGAGAGTTTCAAGAGAACTCTCATGACATGGAAAGGCTTTTTTG |
| shZDHHC4-2 | GATCCGCCACCAACCAGACTACTAATTTCAAGAGAATTAGTAGTCTGGTTGGTGGCTTTTTTG |

**Supplementary Table S3.**

**Target gene sequence of siZDHHC4.**

| **Gene** | **Sense** | **Antisense** |
| --- | --- | --- |
| ZDHHC4-homo-1294 | CCACCAACCAGACUACUAATT | UUAGUAGUCUGGUUGGUGGTT |
| ZDHHC4-homo-1394 | CCGGAACAUUCACUCCCAUTT | AUGGGAGUGAAUGUUCCGGTT |
| ZDHHC4-homo-1450 | CCUUUCCAUGUCAUGAGAGTT | CUCUCAUGACAUGGAAAGGTT |
| AKT-homo-1191 | GACGGGCACAUUAAGAUCATT | UGAUCUUAAUGUGCCCGUCTT |
| AKT-homo-888 | AGGAAGUCAUCGUGGCCAATT | UUGGCCACGAUGACUUCCUTT |
| AKT-homo-816 | GCACUUUCGGCAAGGUGAUTT | AUCACCUUGCCGAAAGUGCTT |
| p70S6K-homo-628 | GUGGAGGAGAACUAUUUAUTT | AUAAAUAGUUCUCCUCCACTT |
| p70S6K-homo-1309 | GUGCCAAUCAGGUCUUUCUTT | AGAAAGACCUGAUUGGCACTT |
| p70S6K-homo-1658 | GCACCUGCGUAUGAAUCUATT | UAGAUUCAUACGCAGGUGCTT |

**Supplementary Table S4.**

**Primer sequences for RT-PCR detection.**

| **Gene** | **Sense** | **Antisense** |
| --- | --- | --- |
| ZDHHC4 | CCACTTGGTGGTGATGTCAG | GTGAATGTTCCGGTGGACTT |
| ALDH1A1 | GCCATAACAATCTCCTCTGCT | CATGGAAACCGTACTCTCCC |
| Nanog | CCCCAGCCTTTACTCTTCCTA | CCAGGTTGAATTGTTCCAGGTC |
| OCT4 | GGGAGATTGATAACTGGTGTGT | GTGTATATCCCAGGGTGATCCT |
| SOX2 | TACAGCATGTCCTACTCGCAG | GAGGAAGAGGTAACCACAGGG |
| CD133 | GAGAAAGTGGCATCGTGCAA | CACGTCCTCCGAATCCATTC |
| β-Actin | CATCCGCAAAGACCTGTACG | CCTGCTTGCTGATCCACATC |

**Supplementary Table S5.**

**The collection of cancer driver genes and palmitoylated proteins identified by mass spectrometry.**

| **Cancer Driver Genes (n=229)** | **Putative Pal Proteins (n=32)** |
| --- | --- |
| TP53, KRAS, PIK3CA, BRAF, LRP1B, KMT2D, APC, ARID1A, KMT2C, PTEN, NRAS, CTNNB1, CDKN2A, IDH1, RB1, ATM, NF1, SMAD4, FAT1, FBXW7, NOTCH1, FAT4, EGFR, ARID2, CREBBP, KDM6A, FAT3, ATRX, SETD2, ERBB2, NFE2L2, SMARCA4, CDH1, SF3B1, VHL, KEAP1, SPOP, PBRM1, EP300, FOXA1, PIK3R1, AR, MAP3K1, TRRAP, ERBB3, GATA3, FGFR3, HRAS, BRCA2, BAP1, STAG2, DDX3X, CDH10, AKT1, STK11, PIM1, BIRC6, MTOR, ESR1, BCOR, NSD1, NCOR1, PTCH1, NOTCH2, KMT2A, RNF43, CTCF, BTG1, CASP8, CDK12, PREX2, KIT, MAP2K4, SPEN, CIC, PTPN11, PTPRB, FAM46C, HLA-A, MYD88, TCF7L2, AXIN1, PPP2R1A, NBEA, ZFHX3, RBM10, FGFR2, FAM135B, MYH9, TBX3, MAP2K1, ELF3, DNMT3A, AMER1, PTPRD, BCL2, CDH11, H3F3A, PRKCB, ARID1B, CHD4, EZH2, ALK, CDKN1A, IDH2, CARD11, RHOA, GNAS, MECOM, MED12, ERCC2, ACVR2A, TGFBR2, SOX9, RUNX1, PPP6C, U2AF1, TSC1, GTF2I, EPHA3, MAX, IRF4, KDR, SGK1, ASXL1, RGPD3, POLQ, PRDM1, CCND1, CUX1, TSC2, NF2, CBFB, ALB, MYCN, PABPC1, SMO, CUL3, P2RY8, CYLD, CDKN1B, GNA11, FLT3, RAC1, MYH11, GSK3β, GNAQ, TNFRSF14, CDC42, BRCA1, SOCS1, RET, SRGAP3, FLT4, RASA2, MEN1, RXRA, B2M, FUBP1,HSPG2, LZTR1, PCBP1, KLF5, CACNA1D, SMAD3, CD79B, PRKD2, SMAD2, RGS7, MEF2B, BCLAF1, LATS1, MYC, RPS6KA3, STAT3, ERBB4, NFKBIE, BCL9L, CHD2, ACVR1, BCL9, EBF1, MET, SP140, XPO1, UBR5, TET2, FN1, PTPRT, TET1, KDM5C, MB21D2, NCOR2, IKZF1, FANCD2, CMTR2, USP6, HIST1H3B, PIK3CB, EIF1AX, ARHGAP35, FGFR1, TBL1XR1, TP63, RUNX1T1, ZNF521, ZFP36L1, PTPRC, ATF7IP, MAPK1, RANBP2, BCR, FAM186A, POT1, NFKB2, PARP4, COL1A1, PDGFRA, AJUBA, NIPBL, CCND3, NIN, BRD7, BCL6, FOXA2, ABL2, DROSHA, RNF213, JAK1 | K1C10, MUC5A, GNAQ, SETBP, ATS1, CDC42, GSK3β, RFA1, Cadm4, EPHB2, SMAD4, NSD1, SMAD3, LRP1, RASA2, SYNE2, SETD2, MEGF8, STAT3, STOX1, Trappc11, VP13B, FRAS1, Mta1, LAMB4, EZH2, GAP43, EZB1, EGFR, AKIB1, STAB1, ZDHHC17 |

**Supplementary Table S6.**

| Residue | Closest | Distance | # HB | # Salt Bridges | # Pi Stacking | # Disulfides | # vdW Clash |
| --- | --- | --- | --- | --- | --- | --- | --- |
| A:1:Met | B:264:Val B:265:Val B:268:Phe | 3.4 A 3.4 A 3.7 A | 0 | 1 | 0 | 0 | 0 |
| A:2:Ser | B:15:Leu | 3.1 A | 0 | 0 | 0 | 0 | 0 |
| A:3:Gly |  |  | 0 | 0 | 0 | 1 | 0 |
| A:4:Arg | B:269:Leu | 3.9 A | 0 | 1 | 0 | 0 | 0 |
| A:5:Pro |  |  | 0 | 0 | 0 | 0 | 0 |
| A:7:Thr |  |  | 0 | 0 | 0 | 0 | 0 |
| A:9:Ser | B:279:Tyr | 3.5 A | 0 | 2 | 0 | 0 | 1 |
| A:10:Phe | B:279:Tyr B:290:Trp | 2.8 A 3.3 A | 1 | 0 | 0 | 0 | 0 |
| A:11:Ala | B:297:Trp | 3.5 A | 0 | 0 | 0 | 0 | 0 |
| A:12:Glu |  |  | 0 | 0 | 0 | 0 | 0 |
| A:13:Ser | B:316:Arg B:314:Val B:315:His | 3.0 A 3.4 A 3.6 A | 0 | 0 | 0 | 0 | 1 |
| A:14:Cys | B:314:Val | 2.7 A | 0 | 1 | 0 | 0 | 0 |
| A:15:Lys | B:311:Glu | 2.6 A | 1 | 1 | 0 | 0 | 2 |
| A:16:Pro | B:312:Pro | 3.2 A | 0 | 0 | 0 | 0 | 0 |

**A:** GSK3β **B:** ZDHHC4

**Supplementary Table S7.**

**Association between ZDHHC4 expression and clinic-pathological characteristics of Glioma patients**

| **Clinical Pathological Parameters** | | **n** | **ZDHHC4** | **X^2^** | **P** |
| --- | --- | --- | --- | --- | --- |
| **Gender** |  |  |  |  |  |
| Male |  | 84 | 65(77.4%) | 0.27 | ＞0.05 |
| Female |  | 41 | 30(73.2%) |  |  |
| **Age (Years)** |  |  |  |  |  |
| ≥45 |  | 61 | 44(72.1%) | 0.94 | ＞0.05 |
| 16-45 |  | 57 | 45(78.9%) |  |  |
| ≤16 |  | 6 | 5(83.3%) |  |  |
| **Grade of Differentiation** |  |  |  |  |  |
| 1-2 |  | 39 | 21(53.8%) | 11.8 | ＜0.01 |
| 3 |  | 28 | 23(82.1%) |  |  |
| 4 |  | 55 | 46(83.6%) |  |  |

**Supplementary Table S8.**

**Association between *p-*GSK3β(Y216) expression and clinic-pathological characteristics of Glioma patients**

| **Clinical Pathological Parameters** | | **n** | ***p-*GSK3β(Y216)** | | **X^2^** | **P** |
| --- | --- | --- | --- | --- | --- | --- |
| **Gender** |  |  |  |  | |  |
| Male |  | 84 | 57(67.9%) | 0.58 | | ＞0.05 |
| Female |  | 41 | 25(61.0%) |  | |  |
| **Age (Years)** |  |  |  |  | |  |
| ≥45 |  | 61 | 43(70.5%) | 1.09 | | ＞0.05 |
| 16-45 |  | 57 | 35(61.4%) |  | |  |
| ≤16 |  | 6 | 4(66.7%） |  | |  |
| **Grade of Differentiation** |  |  |  |  | |  |
| 1-2 |  | 39 | 14（35.9%） | 22.9 | | ＜0.01 |
| 3 |  | 28 | 19（67.9%） |  | |  |
| 4 |  | 55 | 46（83.6%） |  | |  |

**Supplementary Table S9.**

**Association between *p-*STAT3 (Y705) expression and clinic-pathological characteristics of Glioma patients**

| **Clinical Pathological Parameters** | | **n** | ***p-*STAT3 (Y705)** | | **X^2^** | **P** |
| --- | --- | --- | --- | --- | --- | --- |
| **Gender** | |  |  |  | |  |
| Male | | 84 | 45(53.6%) | 0.25 | | ＞0.05 |
| Female |  | 41 | 20(48.8%) |  | |  |
| **Age (Years)** |  |  |  |  | |  |
| ≥45 |  | 61 | 36(59.0%) | 2.72 | | ＞0.05 |
| 16-45 |  | 57 | 25(43.9%) |  | |  |
| ≤16 |  | 6 | 3(50.0%） |  | |  |
| **Grade of Differentiation** |  |  |  |  | |  |
| 1-2 |  | 39 | 12（30.8%） | 9.38 | | ＜0.01 |
| 3 |  | 28 | 16（57.1%） |  | |  |
| 4 |  | 55 | 34（61.8%） |  | |  |
