# Supplementary material for: GSK3β palmitoylation mediated by ZDHHC4 promotes tumorigenicity of glioblastoma stem cells in temozolomide-resistant glioblastoma through the EZH2–STAT3 axis
Source: Oncogenesis. 2022 May 23;11(1):28. doi: 10.1038/s41389-022-00402-w (PMC9126914; doi:10.1038/s41389-022-00402-w)
Supplement: Supplementary file 2 — Authorship Change Agreement [file 41389_2022_402_MOESM2_ESM.pdf]

SPRINGER NATURE

In accordance with Springer Nature Authorship Policy we agree to change the authors of the manuscript as indicated below.

NAME OF JOURNAL: oncogenesis

TITLE OF MANUSCRIPT: GSK3β palmitoylation mediated by ZDHHC4 promotes tumorigenicity of glioblastoma stem cells in temozolomide-resistant glioblastoma through the EZH2 - STAT3 axis

MANUSCRIPT NUMBER: ONCSIS-22-0065

CORRESPONDING AUTHORS NAME: Bing Zhao,Zhiyou Fang and Xueran Chen

PREVIOUS AUTHOR NAMES:

Chenggang Zhao PhD, Huihan Yu MD, Xiaoqing Fan PhD, Wanxiang Niu PhD, Junqi Fan MD, Suling Sun MD, Meiting Gong MD, Zhiyou Fang PhD,* Xueran Chen PhD,#

UPDATED AUTHOR NAMES:

Chenggang Zhao PhD, Huihan Yu MD, Xiaoqing Fan PhD, Wanxiang Niu PhD, Junqi Fan MD, Suling Sun MD, Meiting Gong MD, Bing Zhao, PhD,* Zhiyou Fang PhD,* Xueran Chen PhD,#

CHANGE TO AUTHOR LIST:

Chenggang Zhao PhD, Huihan Yu MD, Xiaoqing Fan PhD, Wanxiang Niu PhD, Junqi Fan MD, Suling Sun MD, Meiting Gong MD, Bing Zhao, PhD,* Zhiyou Fang PhD,* Xueran Chen PhD,#

Print Name	Signature	Date
Chenggang Zhao	Chenggang Zhao	2022-4-15
HuihanYu	Huihan Yu	2022-4-15
XiaoqingFan	Xiaoqing Fan	2022-4-15
WanxiangNiu	Wanxiang Niu	2022-4-15
JunqiFan	Junqi Fan	2022-4-15
SulingSun	Suling Sun	2022-4-15
MeitingGong	Meiting Gong	2022-4-15
BingZhao	Bing Zhao	2022-4-15
ZhiyouFang	Zhiyou Fang	2022-4-15
XueranChen	Xueran Chen	2022-4-15
